# Highly Repeatable Fibrous Crack‐Based Strain Sensor for Multichannel Continuous Tremor Monitoring in Parkinson's Disease and Essential Tremor

**DOI:** 10.1002/advs.76724

**Published:** 2026-08-02

**Authors:** Chiwon Song, Chan Park, Juyoun Lee, Hojoong Kim, Jeongbeom Kang, Byeongjun Lee, Cheoljeong Park, Jungmin Kim, Haran Lee, Yoon Jae Lee, Min‐Kyung Yeo, Woon‐Hong Yeo, Seong J. Cho

**Affiliations:** ^1^ Department of Mechanical Engineering Chungnam National University (CNU) Daejeon Republic of Korea; ^2^ National NanoFab Center (NNFC) Daejeon Republic of Korea; ^3^ Department of Neurology Chungnam National University School of Medicine Daejeon Republic of Korea; ^4^ George W. Woodruff School of Mechanical Engineering Georgia Institute of Technology Atlanta Georgia USA; ^5^ Wearable Intelligent Systems and Healthcare Center (WISH Center) At the Institute for Matter and Systems Georgia Institute of Technology Atlanta Georgia USA; ^6^ School of Electrical and Computer Engineering Georgia Institute of Technology Atlanta Georgia USA; ^7^ Department of Computer Science Georgia State University Atlanta Georgia USA; ^8^ Department of Pathology Chungnam National University School of Medicine Daejeon Republic of Korea; ^9^ Wallace H. Coulter Department of Biomedical Engineering Georgia Institute of Technology and Emory University School of Medicine Atlanta Georgia USA; ^10^ Parker H. Petit Institute for Bioengineering and Biosciences Georgia Institute of Technology Atlanta Georgia USA; ^11^ Korea KIAT‐Georgia Tech Semiconductor Electronics Center (K‐GTSEC) At the Institute for Matter and Systems Georgia Institute of Technology Atlanta Georgia USA

**Keywords:** essential tremor, fibrous crack‐based strain sensor, Parkinson's disease, tremor monitoring system

## Abstract

Objective measurement of tremors is important for assessing the severity and preventing misdiagnosis of Parkinson's disease (PD). To address this demand, this study presents a multichannel, wireless, patch‐type tremor monitoring system that integrates a high‐repeatable fibrous crack‐based strain (FCBS) sensor with a flexible wireless communication circuit. The FCBS sensor, incorporating crack structures, random fiber orientation, junction melting, and polymer penetration within a 3D conductive fiber network, achieves high sensitivity (gauge factor up to 476.89), a fast response time (≈ 6.64 ms), a wide working range (>200% strain), low Young's modulus (≈45 kPa), and excellent repeatability (>100 000 cycles). In addition, this study demonstrates the first clinical application of a flexible strain sensor‐based tremor monitoring system in patients with PD and ET for simultaneous multilocation tremor measurement. Through clinical trials, our system measured tremor indicators (peak amplitude and fundamental frequency), and tremor indicators obtained from multilocation showed improved correlation and statistical significance with tremor scoring in UPDRS III under specific conditions. These results demonstrate the applicability and potential of the flexible strain sensor‐based system as a wearable platform for quantitative and objective tremor measurement.

## Graphical abstract

This study proposes a patch‐type tremor monitoring system based on high‐repeatable fibrous crack‐based strain sensor, capable of simultaneously measuring tremor in multiple locations such as thumb, index, and wrist. Through clinical trials involving Parkinson's disease and essential tremor patients, the proposed system demonstrates its potential as a wearable platform for quantitative and objective tremor measurement.

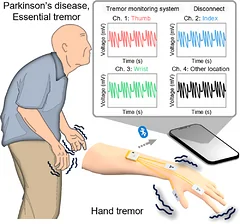

## 1 Introduction

Parkinson's disease (PD) is the second most common neurodegenerative disorder worldwide, and its incidence is steadily increasing with population aging [1]. Among various PD symptoms, tremor is particularly important because it is not only one of the earliest and most troublesome manifestations but also serves as a biomarker [2].

Currently, PD tremor assessment is clinically performed through short‐term subjective evaluations, such as the Movement Disorder Society‐Unified Parkinson's Disease Scale (MDS‐UPDRS) [3]. However, the reliance on the experience of the clinician can lead to several problems. First, this approach can be prone to measurement errors due to inter‐ and intra‐rater variability and short‐term effects [4]. Consequently, the reliability of clinical assessment can be limited. Second, PD can be misdiagnosed due to clinical characteristics that overlap with other diseases, such as essential tremor (ET) [5, 6]. Approximately 24% of patients with early‐stage PD are initially misdiagnosed [7]. Therefore, to assist in more accurate assessment and differential diagnosis, a system capable of quantitative and continuous measurement of tremor characteristics (amplitude and frequency) is required.

Recently, flexible strain sensors have attracted attention for healthcare applications [8, 9, 10]. These sensors can be directly attached to the skin or easily integrated with wearable modules to measure biophysical signals such as pulse [11, 12], respiration [13, 14], and hand movement [15, 16] because of their lightweight, flexible, and stretchable characteristics. Furthermore, these characteristics provide wearer comfort during continuous measurement [17]. Therefore, flexible strain sensors are promising alternatives for pathological hand tremor monitoring. However, previous studies on flexible strain sensors for hand tremor measurement have reported several limitations. First, most studies have demonstrated limited reliability over continuous use due to poor repeatability [18, 19, 20, 21, 22, 23, 24, 25, 26, 27, 28]. Second, slow response times and low sensitivity can degrade tremor signal detection, leading to failure in detecting minute tremors [18, 21, 22, 23, 24, 28]. Third, sensor component materials such as graphene [22], MXene [23], and carbon nanotubes [24, 25, 28] involve expensive production cost [29, 30, 31, 32], which may increase the overall sensor costs. (Table S1) Accordingly, for sensors that require disposable use in healthcare wearable system, such cost increases can exacerbate the economic burden [33]. Also, economic burden acts as a factor that can limit continuous use of healthcare system [34, 35]. Fourth, many previous systems have not been implemented as wearable monitoring systems [18, 19, 20, 21, 22, 23, 26, 27, 28]. Finally, clinical trials on actual patients have not been conducted, with most previous studies being limited to experiments under simulated tremor (ST) conditions [18, 19, 20, 21, 22, 23, 24, 26, 27, 28].

]In this study, we developed a flexible patch‐type hand tremor monitoring system that integrates a high‐repeatability fibrous crack‐based strain (FCBS) sensor with a flexible circuit to overcome these limitations (Figure 1a). The patch‐type form factor is flexible, thin, and lightweight, and can be directly attached to the skin (Figure 1b, Figure S1). As summarized in Table 1, our system exhibits remarkable performance compared with previous flexible strain sensor‐based tremor measurement systems [18, 19, 20, 21, 22, 23, 24, 25]. The primary contributions of our study are as follows: First, the FCBS sensor exhibits superior repeatability (>100 000 cycles), making it suitable for reliable tremor monitoring. Second, it provides a fast response time (≈6.64 ms) and high sensitivity, enabling the detection of minute tremors. Third, the FCBS sensor exhibits low raw material cost (<0.149 USD, Table S2). The reported value consists of only raw material costs, and the total cost may increase when additional costs (e.g., electricity, consumables, and manufacturing equipment) are considered. However, the fabrication process is based on scalable manufacturing processes, such as electrospinning, electroless plating, and laser machining, enabling mass production and thereby reducing manufacturing‐related costs. (Figures S2 and S3). These characteristics allow multilocation tremor measurements without the financial burden of disposable sensors and are advantageous in medical environments in which hygiene and personalization are important (Figure 1c, Figure S4). Fourth, by integrating with multichannel flexible electric circuits that can communicate wirelessly with a portable system via Bluetooth, a hand monitoring system was fabricated as a patch‐type wearable system for simultaneous and real‐time measurement and transmission of hand tremors of various parts, such as the thumb, index finger, and wrist, without restrictions on the wearer's movements (Figure 1d). Finally, we conducted a clinical trial targeting patients with PD and ET using our system.

**FIGURE 1:**
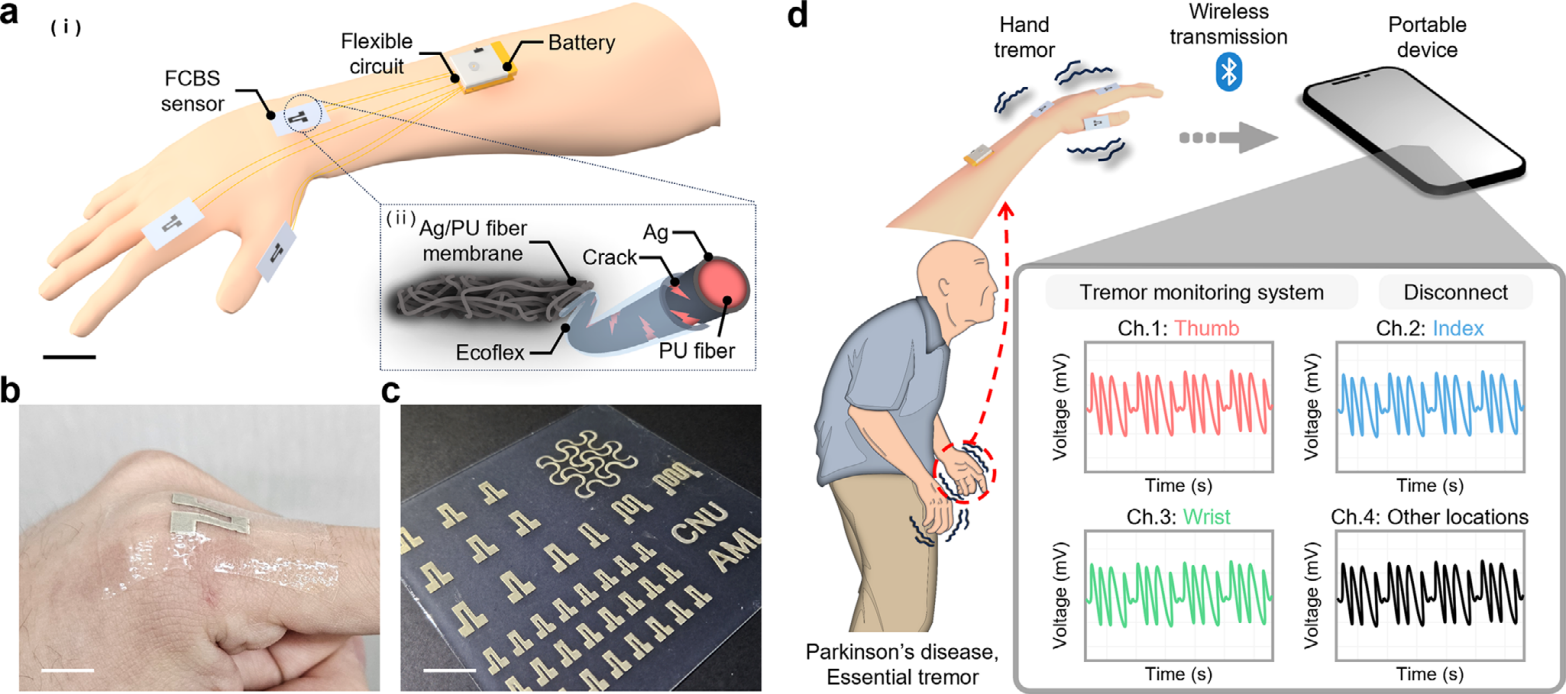
Hand tremor monitoring system. (a) Schematic of hand tremor monitoring system, (b, c) Photograph of FCBS sensor (b) attached to human skin and (c) on a substrate. (d) Schematic of hand tremor monitoring process. Scale bars: (a) 4 cm; (b) 1 cm; (c) 2 cm.

**TABLE 1:** Tremor monitoring system using flexible strain sensor.

				Clinically tested disease				
Repeatability [Cycles]	Response time [ms]	Maximum Gauge Factor	System configuration	PD	ET	ST	Simultaneous measurable system channel	References
100 000	6.64	476.89	Flexible, Wireless communicable, Patch‐type wearable system	O	O	O	4	This works
—	120	13.5	Non‐wearable system	X	X	O	—	[18]
5000	32	—	Non‐wearable system	X	X	O	—	[19]
1000	6.1 ± 1.47	2.37 ± 0.14	Non‐wearable system	X	X	O	—	[20]
200	190	3.74	Non‐wearable system	X	X	O	—	[21]
3000	200	37.9	Non‐wearable system	X	X	O	—	[22]
2000	183	296.8	Non‐wearable system	X	X	O	—	[23]
400	800	37.2	Flexible, Wireless communicable, Patch‐type wearable system	X	X	O	1	[24]
1000	—	4.4	Rigid, Wireless communicable, Glove‐type wearable system	O	X	O	1	[25]
10 000	—	—	Non‐wearable system	X	X	O	—	[26]
—	—	0.71	Non‐wearable system	X	X	O	—	[27]
20 000	16	34.22	Non‐wearable system	X	X	O	—	[28]

To the best of our knowledge, this is the first study to demonstrate multilocation tremor monitoring using flexible strain sensors for measurement of tremor indicators (peak amplitude and fundamental frequency) in patients with PD and ET. In this study, we clinically evaluated a flexible, multilocation tremor monitoring system, confirming its applicability as a wearable platform. Ultimately, our system may enable reliable acquisition of quantitative tremor data, providing a foundation for objective tremor measurement.

## 2 Result and Discussion

### 2.1 FCBS Sensor and Flexible Circuit Performance

The core of our hand tremor monitoring system is the FCBS sensor. The mechanical and electrical properties of the sensor were characterized to confirm its suitability for continuous tremor monitoring. As shown in Figure 2, the FCBS sensor was developed to achieve high sensitivity, repeatability, and flexibility, making it an optimal solution for tremor monitoring in patients with PD. (GF: 17.93 under 60% strain, 57.53 at 60%–140% strain, 476.89 at 140%–200% strain, Young's modulus ≤45.46 kPa, fast response time ≈ 6.64 ms, repeatability >100 000 cycles at 10% strain, measurable frequency ≥50 Hz). The sensor comprises a crack‐induced conductive network using Ag‐coated polyurethane (PU) fibers encapsulated within a soft yet resilient Ecoflex layer. (PU fiber membrane thickness ≈ 33 µm, Ecoflex + PU thickness ≈ 280 µm). The cracks in the conductive network dynamically adjust the electrical resistance in response to mechanical deformation, enabling highly sensitive and quick strain detection.

**FIGURE 2:**
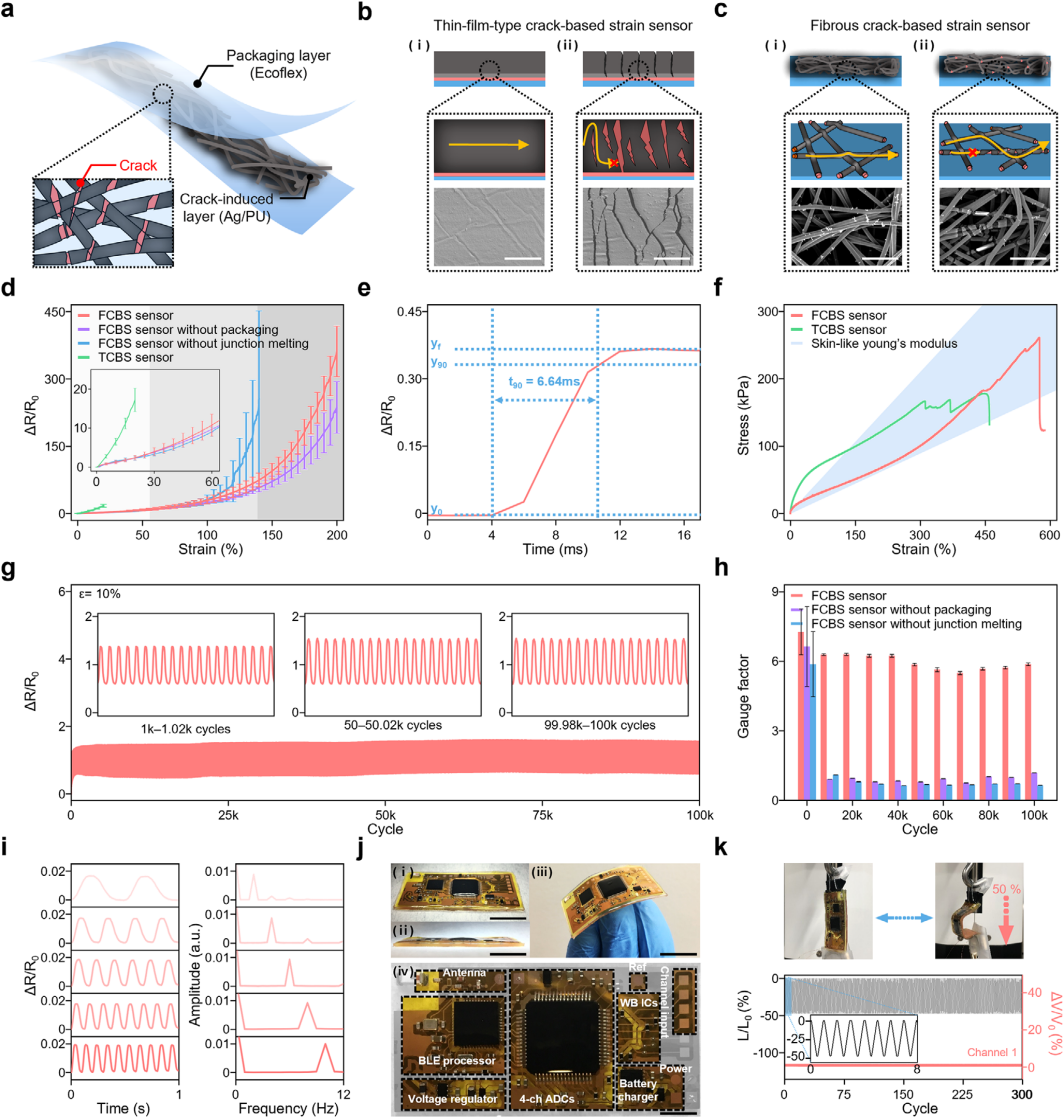
Performance of hand tremor monitoring system. (a) Schematic of FCBS sensor. Mechanism and SEM image of (b) thin‐film crack‐based strain (TCBS) and (c) FCBS sensors.; (i) unstrained state (ii) strained state. (d) Strain–relative resistance curve of TCBS and FCBS sensors. (e) Response time of FCBS sensor under 1% strain (f) Strain–stress curve of TCBS and FCBS sensors. (g) Repeatability test (after 60% pre‐strain, 10% repeat strain) of FCBS sensors. (h) Gauge factor (GF)–cycle graph of FCBS sensor (GF was calculated from 10‐cycle intervals). (i) Measurable frequency of FCBS sensor. (j) Characteristics of soft wireless systems. (k) Cyclic bending test of circuit. Scale bars: (b) 20 µm; (c) 20 µm; (j) (i–iii) 1 cm; (iv) 1 mm.

As shown in Figure 2a, the sensor is structured to enhance mechanical flexibility while maintaining electrical stability. The fibrous architecture ensures reversible crack formation, allowing consistent signal modulation under repetitive strain, which is critical for accurate tremor measurement. In addition, the Ecoflex encapsulation mitigates mechanical fatigue and shields the sensor from environmental degradation, ensuring stable operation over prolonged periods. This continuous reliability is essential for real‐world clinical applications, where sensors must maintain performance without frequent recalibration or replacement. The FCBS sensor is lightweight (0.7 g), ergonomic, and personalized, ensuring comfort for continuous wear.

Also, breathability is a critical characteristic for patch‐type sensors. Although FCBS sensors consist of fiber membrane, breathability is limited because of elastomer layer (Ecoflex mixture) encapsulation. Nevertheless, FCBS sensors showed minimal erythema compared to commercial gel electrodes during 24‐h wearing tests. (Figure S5) The minimal erythema even after 24‐h wearing suggests that FCBS sensor offers a wear comfort in continuous wearing conditions [36]. However, improvement and verification of breathability during prolonged wear conditions remain important for future research. The breathability of these elastomer‐based flexible electronics can be improved through structural engineering such as hole and kirigami using laser patterning [37, 38, 39], which may be applicable strategies for breathability of FCBS sensor in future research.

Figure 2b,c shows the sensing mechanism of the FCBS sensor compared with thin‐film crack‐based strain (TCBS) sensors. Cracks in TCBS sensors are generated and propagated locally in a 2D conductive layer (Figure 2b). Cracks in the FCBS sensor are generated and propagated differently in each fiber strand within the 3D conductive layer of a randomly oriented fiber network (Figure 2c). A more detailed structural configuration of the FCBS sensor is presented in Figure S6.

Under strain conditions, the plated fiber strands are reoriented toward the strain direction and converge parallel to it. Figure S7 and Table S3 show the reoriented fiber images, Gaussian fitting graph, Gaussian fitted mean (center angle), and standard deviation according to the strain level. Both the center angle and standard deviation decreased as the strain increased (13.18° and 27.68° at 0% strain to −0.24° and 11.61° at 180% strain, respectively). During this process, cracks are generated and propagated differently depending on the initial angle, which represents the angle between the fiber and the strain direction at 0% strain (Figure S8, Tables S4 and S5). Importantly, the ratio of the crack length to the fiber length increased more steeply for fibers with an initial angle close to 0°. For example, at 180% strain, the crack length/total fiber length (%) of low‐initial angle fibers (6.5° and 29.8°) is greater than 60.2%, whereas that of high‐initial angle fibers (52.0° and 84.5°) is 24.7%–41.8% (**A detailed description is given** in Section S2.1). Consequently, these fiber orientation‐dependent crack propagation mechanisms enable the FCBS sensor to provide stable and reliable signals across a wider working range than TCBS sensors.

Moreover, the FCBS sensor enhances mechanical and electrical stability by incorporating a composite structural design, including junction melting and polymer penetration. Proper tip‐to‐collector distance control can leave a residual solvent inside the fiber membrane. This residual organic solvent induces junction melting that enhances the mechanical bonding of the fiber network (Figures S6b and S9). This mechanical bonding enhances the structural stability of the fiber and contributes to the electrical signal stability of the FCBS sensor. In addition, the porous structure of the fiber network enables polymer penetration during the encapsulation process (Figures S6c and S10). The penetrated polymer fills the gaps between the fibers, strengthening the bond between the substrate and the fiber layer and preventing fiber sliding and delamination of the conductive layer. This prevention of fiber sliding and conductive layer delamination contributes to maintaining a stable GF of the FCBS sensor (**A detailed description is provided** in Sections S2.2 and S2.3). Under optimized conditions, FCBS sensor performance is described below.

The strain sensitivity and working range of the optimized FCBS sensor were evaluated to determine its effectiveness in detecting tremors of varying intensities. Strain sensitivity, typically expressed as the GF, is a crucial parameter for detecting small‐scale biomechanical motions, such as tremors. A higher GF allows for more precise detection of minute strain variations, which is essential for capturing low‐amplitude tremors that typically occur in the early PD stages. The working range of a sensor defines the maximum strain that it can endure while maintaining functionality, ensuring that it remains effective even under increased voluntary hand movements that occur during daily activities. As illustrated in Figure 2d, the FCBS sensor exhibits GF values of ≈ 17.90 in the low‐strain region (0%–60%) and ≈57.53 and ≈476.89 in the high‐strain region (60%–140% and 140%–200%, respectively) while maintaining stable performance up to 200% strain. The high GF in the low‐strain region enables the sensor to detect subtle tremors, which are one of the earliest and most prominent symptoms of PD. Because these tremors typically exhibit a low displacement, a sensor with high‐strain sensitivity is required to accurately capture their presence and intensity. In addition, the extended working range up to 200% strain ensures that the sensor remains operational even when exposed to increased hand movements, thereby preventing signal loss or mechanical failure during natural hand motions. The combination of high sensitivity in the low‐strain region and an extended working range makes the FCBS sensor highly effective for tremor measurement. By maintaining stable performance across a wide strain range, the sensor can accurately record pathological tremors and voluntary movements, thereby contributing to the reliability of our tremor measurement platform.

A fundamental requirement for accurate tremor monitoring is a fast response time to capture rapid biomechanical deformations. As shown in Figure 2e, the FCBS sensor exhibits an ultra‐fast response time ≈ 6.64 ms, which is significantly faster than the Nyquist frequency limit of 41.7 ms (24 Hz) required to accurately detect tremors up to 12 Hz. Parkinsonian tremors mostly fall within the 4–6‐Hz range at rest [40, 41], and may shift at slightly higher frequencies during postural state [42], partially overlapping with essential tremor (ET), which typically reported to span a broader frequency range 4–12‐Hz [43, 44, 45]. Therefore, maintaining a response time well below this threshold ensures that the sensor can faithfully record both low‐ and high‐frequency tremor signals without aliasing or signal degradation. This rapid response is attributed to the instantaneous crack opening and closing mechanism in the Ag‐coated PU fiber network, which allows for immediate resistance modulation in response to external strain. Unlike conventional strain sensors that rely on bulk deformation or slow material relaxation, the crack‐based mechanism ensures high‐speed strain detection with minimal latency. By combining an ultra‐fast response time and real‐time strain detection, the FCBS sensor ensures that even the most transient tremor fluctuations are captured with precision. This makes it highly suitable for continuous, real‐time tremor monitoring, providing reliability for tremor measurement.

For effective tremor measurement in hand, a wearable strain sensor must exhibit mechanical compatibility with the human skin and soft tissues. Unlike rigid or high‐modulus materials, the human skin is highly deformable and compliant, meaning that any sensor applied to the skin must be able to stretch and bend without causing discomfort or affecting natural movement. If a sensor is too rigid, it may hinder natural biomechanics, reduce signal accuracy due to poor skin conformity, or even lead to sensor detachment over extended use. Therefore, evaluating the stress–strain characteristics of the sensor is essential to ensure comfortable and stable continuous operation in real‐world applications. As shown in Figure 2f, the FCBS sensor exhibits a low Young's modulus of ≈45 kPa, which closely aligns with the elastic moduli of the human finger skin (≈30 kPa) and dorsal forearm skin (≈69 kPa) [46, 47]. In addition, the FCBS sensor exhibits a Young's modulus similar to that of the skin, whereas the Young's modulus of the TCBS sensor (114.56 kPa) exceeds that of the skin and is 2.45 times higher than that of the FCBS (46.70 kPa) at below 60% strain (Figure S11). This similarity ensures that the FCBS sensor can naturally conform to the skin's surface, adapting to subtle hand deformations and muscle contractions without generating excessive mechanical resistance or discomfort. In addition, this low Young's modulus enables the FCBS sensor to adhere firmly to the skin and flex well with skin movements, ensuring high skin compliance.

For continuous reliability, a strain sensor must exhibit both repeatability and durability, ensuring stable performance under prolonged cyclic deformation. In real‐world applications, wearable sensors are subjected to continuous mechanical stress, including repetitive stretching, bending, and compression. A sensor exhibiting significant signal drift or degradation over time may fail to provide consistent and reliable tremor data, thereby compromising its usefulness in clinical and everyday monitoring. Therefore, assessing repeatability and durability under cyclic strain is essential to validate the suitability of our sensor for continuous tremor monitoring. As shown in Figure 2g, the FCBS sensor was subjected to 100 000 cycles of 10% strain at a strain rate of 0.3 mm/s to evaluate its electrical stability under prolonged use. Throughout the test, the resistance change (ΔR/R_0_) remained highly consistent, indicating minimal performance degradation. Minor fluctuations observed within the first 1000 cycles are attributed to microstructural stabilization of the conductive cracks, a common phenomenon in crack‐based strain sensors because the initial crack alignment adjusts under repeated strain. Once this stabilization phase was completed, the FCBS sensor exhibited negligible signal drift, confirming its exceptional durability and continuous reliability. This remarkable durability contributed to the stable sensitivity of the FCBS sensor, enabling it to maintain 80.73% of its initial sensitivity after 100 000 cycles of repeat tensile test (Figure 2h). Therefore, after 1000 cycles of sensor aging, the FCBS sensor exhibits outstanding electrical signal stability compared with the TCBS sensor (Figure S12).

Because tremor monitoring requires capturing oscillatory motion within a specific frequency range, the frequency response of the FCBS sensor was analyzed using fast Fourier transform. A sensor should be able to detect tremor signals across a wide range of frequencies, because different neurological conditions exhibit distinct frequency patterns. Parkinsonian tremors typically vibrate within the 4–6‐Hz range at rest state [40, 41], and can slightly widen to higher frequencies during postural state [42]. This frequency range partially overlapping with essential tremor (ET), which typically reported to span a broader frequency range 4–12‐Hz [43, 44, 45]. If a sensor lacks sufficient frequency resolution, it may fail to distinguish between pathological tremors and voluntary movements, resulting in inaccurate measurement of tremor indicators (amplitude and frequency). As shown in Figure 2i, the FCBS sensor effectively detects oscillatory signals at 2, 4, 6, 8, and 10 Hz, confirming its high sensitivity across the clinically relevant tremor frequency range. In addition, vibration measurement test results using a vibration machine demonstrate that the FCBS sensor can accurately measure up to 50 Hz, exceeding the frequency range of various pathologic tremors such as PD, ET, and dystonic tremors that occur between 1 and 25 Hz (Figure S13) [48]. The distinct periodic variations in resistance highlight the ability of the sensor to capture high‐fidelity tremor signals in real time without significant noise or signal distortion. This ensures that even subtle variations in tremor characteristics can be accurately recorded, enabling precise differentiation between PD tremors, ETs, and normal physiological movements.

For real‐time tremor monitoring, a wearable sensor must not only detect hand tremors with high sensitivity but also be capable of wirelessly transmitting the acquired data for remote analysis. A wired system may impose mobility constraints and cause discomfort for continuous use, making it impractical for continuous monitoring. To overcome these limitations, the FCBS sensor was integrated with a flexible circuit designed to enable multichannel signal processing and wireless communication (Figure 2j, Figure S14). The developed electronic module is lightweight (1.5 g) and compact, ensuring that the system can be comfortably worn for extended periods without causing physical strain or interfering with natural movement. Furthermore, the multichannel configuration allows the system to simultaneously acquire signals from multiple sensors placed at key anatomical locations, such as the thumb, index finger, and wrist (maximum four positions), as shown in Figure 2j. This enables comprehensive tremor measurement, because different parts of the hand may exhibit varying tremor intensities depending on disease progression and patient‐specific motion patterns. By providing a holistic view of tremor activity, this configuration enhances the accuracy and clinical relevance of the acquired data. Because wearable systems are constantly subjected to mechanical stress, the durability of the flexible circuit must be thoroughly validated to ensure continuous reliability. As shown in Figure 2k, the circuit was subjected to 300 cycles of 50% bending strain to simulate repetitive flexing and stretching that occur during daily activities. Despite these mechanical deformations, the system maintained stable performance, with signal fluctuation remaining below 1.5%. This confirms that the incorporated flexible circuit can endure prolonged mechanical stress without degradation, ensuring consistent electrical characteristics over extended use. To further assess the wireless transmission capability of the system, the received signal strength indicator was measured at varying distances. As shown in Figure S15, the system maintained stable wireless communication with portable devices, achieving a data transmission rate of 781 bytes/s. This level of robust wireless connectivity ensures that tremor data can be reliably transmitted to a nearby mobile device or clinical monitoring system in real time. In addition, the circuit can provide continuous measurements for up to 30 h, depending on the battery capacity and number of measurement channels (Figure S16). As shown in the above results, our system has a combination of ultra‐fast response time, exceptional mechanical resilience, and stable wireless connectivity. Therefore, our tremor monitoring system shows potential as a wearable tremor‐recording device for quantitative tremor phenotyping.

### 2.2 Tremor Measurements in Various Movements Using FCBS Sensor‐Based Hand Tremor Monitoring System

In Section 2.1, we have demonstrated that the FCBS sensor can accurately measure vibrations in a range of 2–50 Hz (Figure 2i, Figure S13). This vibration sensing capability suggests that the FCBS sensor can effectively detect tremor symptoms of movement disorders that occur in different frequency ranges, such as PD (4–8 Hz) [40, 41, 42] and ET (4–12 Hz) [43, 44, 45]. However, the ability of our system to measure hand tremors during various daily life movements requires further verification.

To quantitatively verify this ability, FCBS sensors were attached to a subject's thumb, index finger, and wrist and the flexible circuit located on the upper forearm. A muscle simulator was also placed in the flexor digitorum profundus and flexor pollicis longus muscles to generate tremors of 8–10 Hz in the thumb, index finger, and wrist (Figure S17).

The subject then performed two types of movement conditions: static and dynamic. Static movement conditions that have minimal body movement, including (1) rest, which is performed while sitting on a chair with the forearm resting on an armrest, and (2) postural, which is performed while sitting with both arms extended forward (Figure 3a‐i, ii). Dynamic movement conditions involving active body movement, including (1) shoulder shaking, which is performed sitting with both arms extended forward while repetitively moving the arms up and down, (2) walking, (3) running, and (4) stair descent (Figure 3a‐iii–vi). From the data measured for each movement, 10 s of data were extracted from the section in which the tremor was observed. PSD estimation was then performed on this segment using Equation (S1).

**FIGURE 3:**
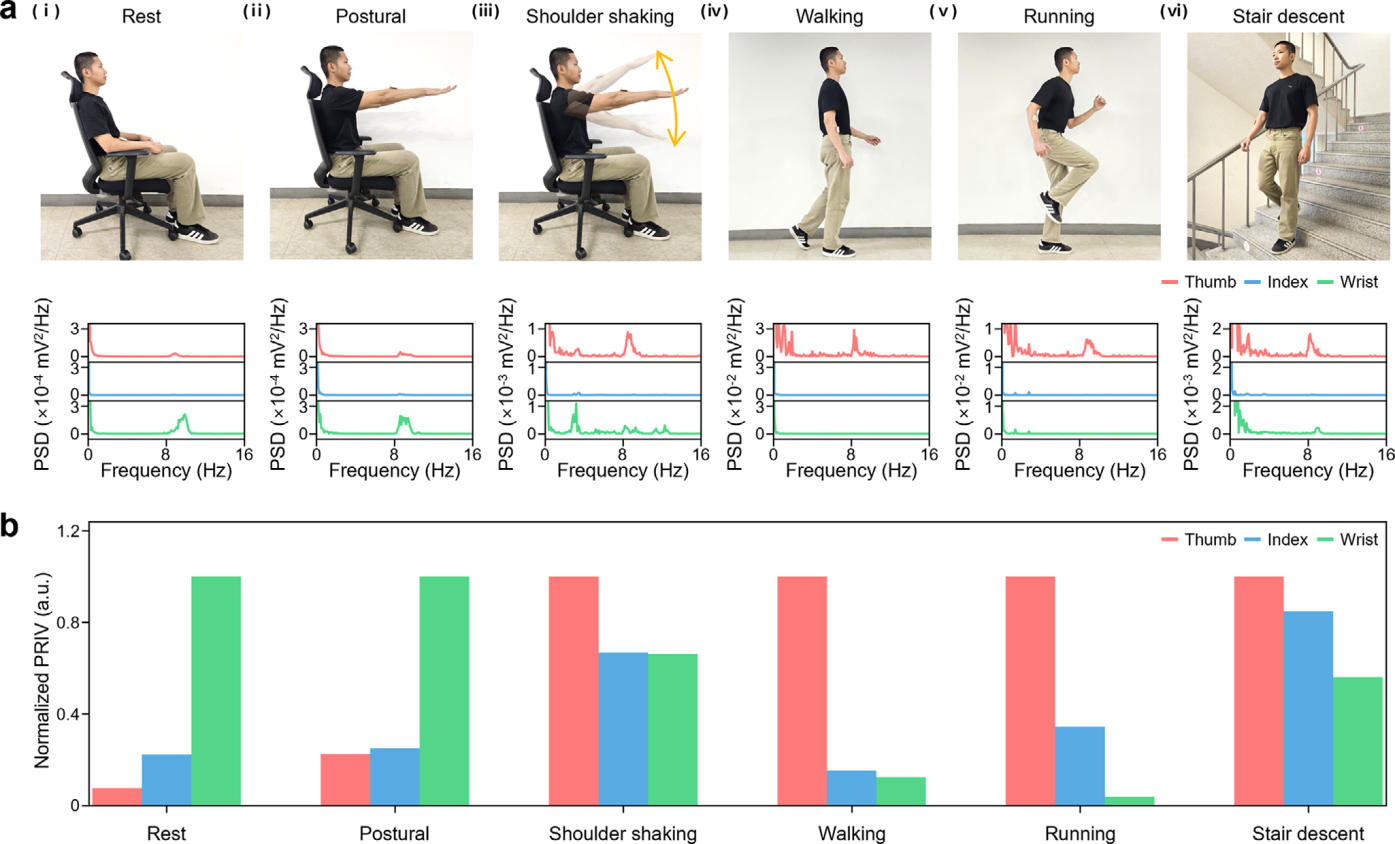
Tremor measurements in daily life movements using FCBS sensor‐based hand tremor monitoring system. (a) Measurement posture and power spectral density of multilocation: (i) rest tremor, (ii) postural tremor, (iii) shoulder shaking, (iv) walking, (v) running, and (vi) stair descent. (b) Normalized power spectral density (PSD) ratio of involuntary to voluntary movement.

The PSD of the tremor signals simulated during each movement collected by our system are shown in Figure 3a. The results confirm that our system effectively detects not only tremor signals but also body movement signals. Under both static and dynamic movement conditions, distinct PSD peaks were observed in at least one and up to three locations, namely, the thumb, index finger, and wrist, in a range of 8–10 Hz, which is estimated to be the tremor frequency range. Furthermore, distinct PSD peaks were observed in the low‐frequency range below 0.5–3 Hz, which is presumed to reflect body movements [49, 50, 51]. This result suggests that our system detects tremors even under various movement conditions.

However, because the PSD peak values varied across the three measurement locations in each movement, it was difficult to determine which location could more effectively detect tremors in specific movements. A quantitative comparison was performed to determine which location more effectively detects tremors under specific movements by defining the PSD ratio of involuntary to voluntary movement (PRIV), as shown in Equation (S2). In the equation, involuntary movement refers to tremor‐related PSD in the 8–10‐Hz range, and voluntary movement corresponds to movement‐related PSD in the 0.5–3‐Hz range. Subsequently, max normalization was performed using the location (among the thumb, index finger, and wrist) with the highest PRIV for each movement, as shown in Equation (S3) and Figure 3b.

During static movements (rest, postural), the wrist exhibited the highest normalized PRIV, up to 13.3 times higher than that of the thumb and index finger. The specific values were as follows: at rest, the PRIV values for the thumb, index finger, and wrist are 0.075, 0.221, and 1.000, respectively; in a postural state, the values are 0.225, 0.249, and 1.000, respectively. In contrast, during dynamic movements (shoulder shaking, walking, running, and stair descent), the thumb exhibited the highest normalized PRIV value, up to 26.3 times that of the index finger and wrist. The specific values are as follows: shoulder shaking (thumb: 1.000; index finger: 0.668; wrist: 0.661), walking (thumb: 1.000; index finger: 0.152; wrist: 0.123), running (thumb: 1.000; index finger: 0.345; wrist: 0.038), and stair descent (thumb: 1.000; index finger: 0.848; wrist: 0.560).

These results, which demonstrate that the dynamic movement measured at the thumb has higher values than that measured at the other locations, are consistent with the passive arm swing mechanism described by Pontzer et al. [52], where the arm acts as a coordinated mass damper that reduces upper body and head rotation. For example, during static movements, there are no factors other than the simulated tremor that induce arm movement; thus, the tremor signal can be strongly detected in the wrist, which has a high degree of freedom. In contrast, during repetitive arm‐shaking movements or movements in which the arm naturally moves to compensate for lower body angular momentum, the arm acts as a mechanical damping mechanism. Consequently, tremor signals at the wrist decrease, whereas distal parts, such as the thumb and index finger, may exhibit stronger tremor signals. This interpretation is consistent with the comparison results of PRIV under postural and shoulder shaking conditions, as well as during walking and running.

Specifically, under postural conditions, PRIV was highest at the wrist, whereas under shoulder shaking conditions, where shoulder movement is added to postural conditions, the PRIV at the distal part of the thumb was relatively higher than that at the wrist. Furthermore, a comparison of the PRIV between walking and running shows that the PRIV at the index finger increased from 0.152 to 0.345 during running, whereas it decreased from 0.123 to 0.038 at the wrist. This suggests that tremor signals became more prominent at the distal part and less prominent at the wrist as upper body movement became more intense. These results suggest that increased linkage stiffness on the upper limb reduces tremor signals at the wrist while enhancing signals at the distal part. This highlights the importance of performing measurements at multiple locations simultaneously rather than relying on a single location.

These research findings demonstrate the potential of FCBS sensor‐based systems for effective tremor movements during various daily movements. Furthermore, by comparing and analyzing the PRIV for each movement across the thumb, index finger, and wrist, we showed that simultaneous measurements of multiple locations may be suggested as a compensatory approach to location‐dependent variability of tremor measurement.

### 2.3 Tremor Measurement in Patients With PD or ET Using FCBS Sensor‐Based Hand Tremor Monitoring System

This section aims to explore the application of our hand tremor monitoring system in patients with PD and ET. Hand tremor data were collected from the thumb, index finger, and wrist simultaneously during rest and an outstretched posture that induces postural tremor, and statistical analyses were performed (Figure S18). In addition, to evaluate the necessity of simultaneous multilocation measurement, we included an “all location” indicator in the analysis, which represents the average amplitude and frequency of hand tremors measured at the thumb, index finger, and wrist. The collected hand tremor signals were analyzed after processing, as described in Figure S19. The patients analyzed in this study were clinically diagnosed with PD and ET by a neurologist specializing in neurodegenerative diseases at a tertiary hospital in accordance with the Movement Disorder Society (MDS) diagnostic criteria [53], and their tremor was scaled by UPDRS motor III. Additionally, patients diagnosed with both PD and ET were classified into the PD group if clinically rest tremor was confirmed, and the ET group if essential tremor and postural tremor were confirmed regardless of medication status. We excluded patients with clinically enhanced physiologic tremor and severe cases due to the small sample size. Most of PD patients were tremor‐dominant PD (TrD‐PD). One participant was mixed type PD. And there was no rigidity‐dominant PD (RD‐PD). Consequently, both groups had comparable sample sizes (Total patients: 21, PD group: 11, ET group: 10), and there were no statistically significant differences in sex (*p* = 0.863), age (*p* = 0.918), H & Y stage (*p* = 0.769), tremor laterality site (*p* = 0.197), medication status (*p* = 0.251) (Mann–Whitney U test). Therefore, the two groups were considered valid comparison populations. In this study, we recruited patients whose dominant symptom was hand tremor, However, anatomical information regarding locations where tremor dominant such as the thumb, index, and wrist was not collected. Detailed information about the patients who participated in the experiment (group, participants, sex, age, diagnosis, H & Y stage, symptom duration in PD, treatment duration in ET, tremor laterality site, medication status, medications) is presented in Table S6.

We conducted a Mann–Whitney U test to examine of the hand tremor data collected using our system in quantitatively comparing the differences in tremor characteristics between the two disease groups. The results are described in **manuscript** Section 2.3.1, “Statistical comparison of tremor parameters between PD and ET” (Figure 4a–c). In addition, Spearman's rank correlation analysis was performed between the Tremor score (TS)s of the MDS‐UPDRS Part III assessed by a neurologist and tremor indicators (peak amplitude (PA) and fundamental frequency (FF)) measured by our system. The results are described in **manuscript** Section 2.3.2, Statistical correlation analysis of tremor indicators in PD and ET.

**FIGURE 4:**
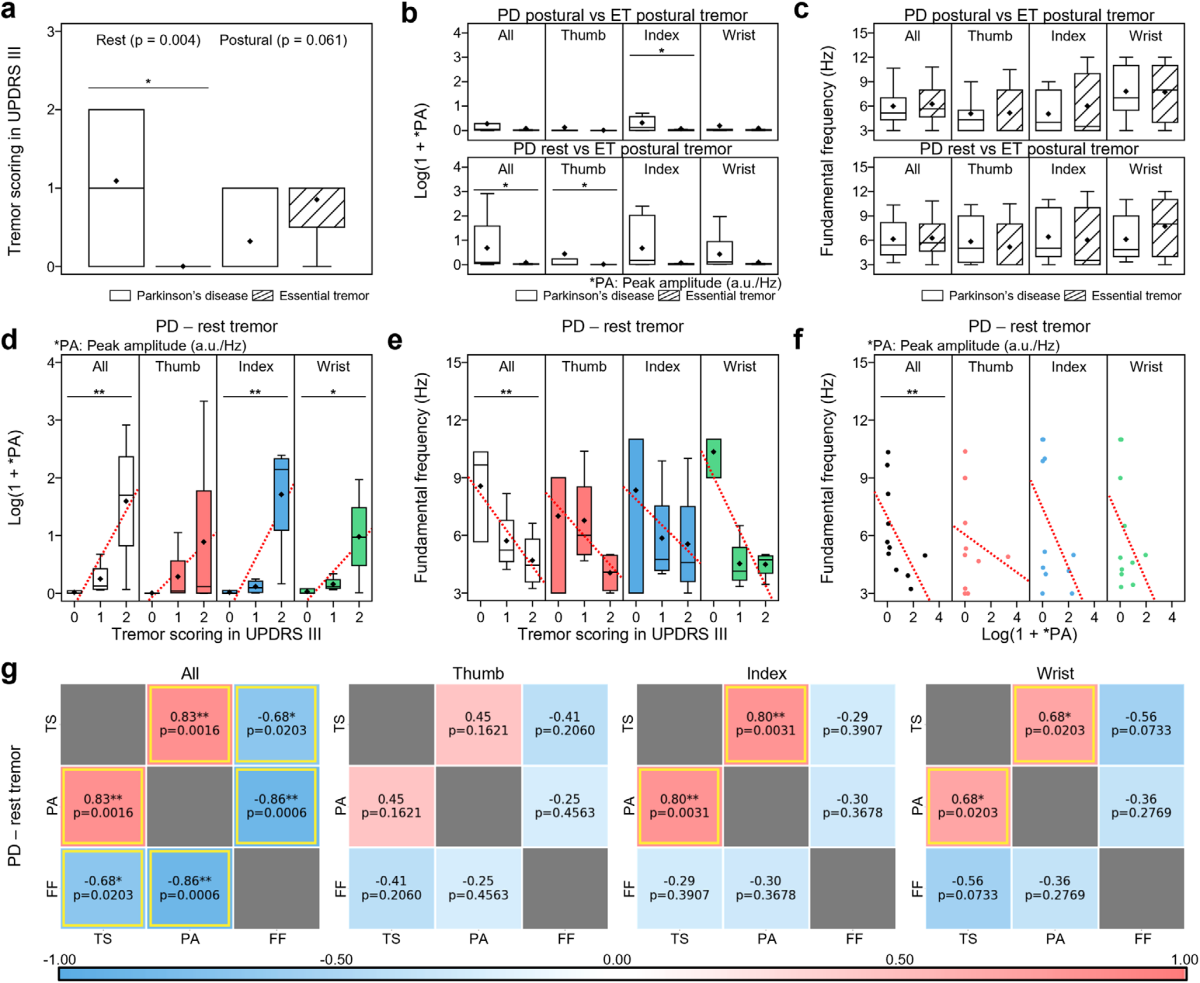
Data distribution analysis of PD and ET (significance level (**): *p* < 0.01, significance level (*): *p* < 0.05): (a) Tremor scoring in UPDRS III; (b) peak amplitude (top: PD postural tremor vs. ET postural tremor; bottom: PD rest tremor vs. ET postural tremor); (c) fundamental frequency (top: PD postural tremor vs. ET postural tremor; bottom: PD rest tremor vs. ET postural tremor). Correlation analysis of tremor parameters during rest tremor in patients with PD (red line represents the linear regression line). (d) Tremor scoring in UPDRS III, peak amplitude; (e) tremor scoring in UPDRS III, fundamental frequency; (f) peak amplitude, fundamental frequency. (g) Correlation matrix of PD rest tremor between tremor indicators and tremor scoring in UPDRS III. (TS: tremor score; PA: peak amplitude; FF: fundamental frequency) Detailed statistical analysis results, such as sample size, exact *p*‐value, and 95% confidence intervals, are provided in Tables S7–S9.

This section presents quantitative indicators obtained from a patch‐type wearable system based on strain sensors that are able to provide objective and quantitative tremor measurement. Specifically, the simultaneous measurement of hand tremors at multiple locations (thumb, index finger, and wrist) using our system and a statistical comparison of tremor characteristics between PD and ET based on these data are considered a distinguishing feature from previous studies. Therefore, by investigating the quantitative characteristics of tremors specific to each disease and suggesting a basis for future studies, this analysis may contribute to future system research aimed at distinguishing between PD and ET.

#### 2.3.1 Statistical Comparison of Tremor Indicators Between PD and ET

Analysis of the tremor indicator (amplitude, frequency) collected by the hand tremor monitoring system between patients with PD and ET was performed. We statistically analyzed the PA and FF data collected simultaneously from the thumb, index finger, and wrist. Recruited patients with PD and ET exhibited typical tremor characteristics. Patients with PD exhibited mild rest tremor, and some patients exhibited postural tremor (rest tremor median: 1.000; postural tremor median: 0). In contrast, patients with ET exhibited no rest tremor but mild postural tremor (rest tremor median: 0; postural tremor median: 1.000). (Figure 4a) These results suggest that the patient population of the two groups adequately reflects the general characteristics.

In addition, differences in each tremor indicator (PA or FF), which were collected from multiple locations (thumb, index finger, wrist, and their average: all), were analyzed between PD and ET under postural tremor conditions, as well as between PD rest tremor and ET postural tremor conditions (Figure 4b,c, Table S7). Rest tremors were evaluated only in PD patients, because ET patients did not exhibit rest tremor based on clinical assessment. Therefore, the signals collected from ET patients in the rest condition were not considered as rest tremor and were used as baseline reference (baseline state). Figure 4b illustrates two graphs. The graph on the top row shows the contrast PA distribution of postural tremors between PD and ET. The graph on the bottom row compares the PA distribution between PD rest and ET postural tremors. Under postural tremor conditions, the PA of patients with PD was statistically greater than that of patients with ET only in the index finger location (PD postural: 0.117; ET postural: 0.006; *p* < 0.05), and no statistical differences were observed in the remaining locations (Figure 4b, **top**). When comparing between PD rest and ET postural tremors that representative tremor characteristics of each disease, the PA values of PD rest tremor and ET postural tremor were statistically different across all locations (PD rest: 0.087; ET postural: 0.014; *p* < 0.05) and thumb (PD rest: 0.010; ET postural: 0.001; *p* < 0.05) locations (Figure 4b, **bottom**). These tendencies are consistent with the prior studies (Henderson et al. [54], Jankovic et al. [55], and Weede et al. [56],) reported that PD postural tremor has a higher amplitude than ET postural tremor, in line with previous study of Henderson et al. [54], reported that PD rest has a higher amplitude than ET postural tremor. As reported in previous clinical studies, early‐stage PD tremor exhibits a distal‐dominant pattern [57], whereas ET tremor is observed to be proximal‐dominant [58]. Therefore, to detect tremor while reflecting the characteristics of each disease, data collection from multiple locations may be required. Indeed, as the above results, the peak amplitude of the tremor showed a statistically significant difference not only when considering specific locations but also when considering all locations. This statistically significant result at the thumb location may be attributed to the tremor‐dominant site in early‐stage PD and ET patients, as reported in previous clinical studies [57, 58]. Furthermore, the statistically significant result at both all and thumb locations suggests that multilocation measurements may be required for comprehensive data collection when recording quantitative tremor indicators using our tremor monitoring system.

Figure 4c presents the distribution of FF obtained at each measurement location (thumb, index finger, wrist, and their average: all). The top row shows the FF distribution of postural tremor between patients with PD and ET (Figure 4c, **top)**, and the bottom row shows the FF distribution between patients with PD rest and ET postural tremors (Figure 4c, **bottom)**. At every location (i.e., thumb, index finger, and wrist), no statistically significant differences were observed in FF, suggesting that FF may have limited meaning indicator for distinguishing tremor characteristics between the two diseases. However, under postural tremor conditions, the median value across all locations (averaging thumb, index finger, and wrist) was higher for patients with ET than for patients with PD (PD: 5.167 Hz; ET: 5.667 Hz). In addition, when comparing PD rest and ET postural tremors, the median value at all locations (PD rest: 5.389 Hz; ET postural: 5.667 Hz) and wrist (PD rest: 4.857 Hz, ET postural: 8.000 Hz) locations were higher for ET postural tremor than for PD rest tremor. This tendency aligns with previous studies that indicate that tremor frequencies occur at higher frequencies in patients with ET than in patients with PD, as measured and quantified by our system [59].

The consistency of our results with previous clinical studies suggests applicability of our tremor monitoring system for the quantification of tremor indicators (PA and FF) in patients with ET and PD. Furthermore, differences in statistical significance of PA depending on the measurement locations may reflect the inter‐patient variability in tremor location, as well as differences in the dominant tremor locations by disease. Also, averaging of measurement signals that are collected from individual locations may function as a complementary measurement approach to mitigate variance of location‐dependent and random errors (**a detailed description is provided** in Section S1.6). In this context, simultaneous measurements at multiple locations show potential as a complementary approach to collect comprehensive tremors. However, considering the limitations of this analysis, including the lack of statistical significance in some results, the limited sample size, and the absence of cohort stratification by motor subtype, the observations described above should be interpreted with caution, and further validation may be warranted.

#### 2.3.2 Statistical Correlation Analysis of Tremor Indicators in PD and ET

As shown in the previous section, the quantified amplitude and frequency values measured in patients with PD and ET may provide meaningful information for capturing tremor characteristics. To further investigate the relationship between tremor indicators (tremor amplitude, frequency) and the TS assessed by clinicians using UPDRS III, correlation analysis was conducted in this section.

Figure 4d–g, Figure S20, and Tables S8 and S9 show Spearman's rank correlation analysis conducted to analyze the relationship between the TS (UPDRS III) evaluated by the clinician and the hand tremor indicators measured simultaneously at multiple locations. The correlation analysis was performed separately according to tremor type (rest tremor, postural tremor) and measurement location (all, thumb, index finger, wrist). We analyzed the correlation between the tremor indicators (PA, FF) under each condition (disease group (PD, ET) × tremor type (rest tremor, postural tremor) × measurement location (all, thumb, index finger, wrist)) with the clinician's TS. TS in UPDRS III of ET in rest condition was recorded as 0 for all patients. Therefore, ET in rest condition was excluded from the analysis (Figure S20).

The results of the correlation analysis between the TS in UPDRS III and PA demonstrate that PA is correlated with TS in UPDRS III and that simultaneous multilocation measurement may provide an additional approach in addition to single‐location measurements. This correlation was particularly evident in rest tremors in patients with PD (Figure 4d–g). A significant positive correlation between the TS in UPDRS III and PA was observed in all (ρ = 0.828, *p* = 0.002), index finger (ρ = 0.799, *p* = 0.003), and wrist (ρ = 0.684, *p* = 0.020) locations, with the highest correlation coefficient and lowest *p*‐value observed at all locations, which average the data obtained at thumb, index finger, and wrist (Figure 4d,g). These results suggest a statistical correlation between the TS in UPDRS III, which is subjectively rated by clinicians, and the tremor amplitude quantitatively measured using our system, demonstrating that PA is correlated with TS assessed by clinicians. In addition, the higher correlation coefficient observed in all locations, which considers every measurement location simultaneously may capture comprehensive tremor characteristics, compared with the data obtained at single locations.

Similarly, in patients with PD with rest tremors, both the correlation between the TS in UPDRS III and FF and the correlation between PA and FF were statistically significant in all locations. These results suggest that fundamental frequency may be correlated with TS and may support the use of multiple location measurements (Figure 4e–g). A statistically significant negative correlation between the TS in UPDRS III and FF was observed only in the all location group (ρ = −0.684, *p* = 0.020), and a strong negative correlation between PA and FF was also observed only in the all location group (ρ = −0.864, *p* = 0.001). In addition, PA, which quantifies the tremor amplitude, exhibited a significant positive correlation with the TS in UPDRS III, whereas FF exhibited a negative correlation. These results are consistent with those of previous studies that demonstrated that amplitude and frequency, respectively, increases and decreases with increasing tremor severity in patients with PD and ET [60, 61, 62]. These results suggest that multilocation analysis may have the potential to reveal statistical significance relationships not observed in single‐location analysis.

In the postural tremor of patients with PD, a significant negative correlation (ρ = −0.825, *p* = 0.002) was observed between the TS in UPDRS III and FF measured at all locations, similar to the rest tremor (Figure S20a). However, in the postural tremor of patients with PD, no overall statistical correlation was observed among the TS in UPDRS III, PA, and FF obtained at the thumb, index finger, and wrist locations. In contrast, in the postural tremor of ET patients, a statistically significant positive correlation was observed between the TS of UPDRS III and PA. Specifically, a significant correlation was prominent in all locations (ρ = 0.884, *p* = 0.001) and wrist locations (ρ = 0.651, *p* = 0.041) (Figure S20b). These observed correlations between tremor indicators and TS in UPDRS III of patients with PD and ET, together with the higher correlation coefficients and lower *p*‐values of the multilocation measurement approach compared to the single‐location approach, suggest that multilocation may provide comprehensive tremor information. This improvement in the correlation coefficient and statistical significance of all locations may be the result of mitigating the variance of error through averaging. (**A detailed description is given** in Section S1.6) Most of the major correlations reported in this study were found to be statistically significant even after *p*‐values were corrected using the Benjamini–Hochberg method (Tables S10 and S11). These results provide additional evidence to support the interpretation of our clinical findings. However, rather than replacing a single location‐based analysis, it should be regarded as a complementary approach that provides additional interpretive value. Furthermore, considering the limitations of this analysis, including the lack of statistical significance in some results and the limited sample size and the biased distribution of TS in UPDRS III under postural tremor conditions, the observations described above should be interpreted with caution and further validation may be required.

## 3 Conclusion

In this study, we developed a patch‐type hand tremor monitoring system by integrating a high‐performance FCBS sensor with a wireless flexible electronic circuit. The developed system addresses the limitations of conventional tremor monitoring approaches by offering continuous, multilocation, and skin‐conformal monitoring in a lightweight, patient‐friendly form factor.

The FCBS sensor, which was fabricated by introducing crack structures on a conductive layer‐deposited fiber surface and applying fiber network junction melting and polymer encapsulation strategies, exhibited superior performance compared with a conventional 2D crack sensor. Specifically, high repeatability (>100 000 cycles), a high GF (476.89 at 140%–200%), a wide working range (maximum 200%), a skin‐like Young's modulus (approximately 45.46 kPa), and a fast response time (≈ 6.64 ms) contribute to the precise measurement of tremors occurring in the human hand, such as PD and ET.

The FCBS sensor exhibited exceptional repeatability, high sensitivity, a wide strain range, and skin‐like mechanical properties, enabling the accurate detection of tremors associated with PD and ET. When integrated with a flexible multichannel circuit, the resulting patch‐type system enabled simultaneous and reliable measurement of tremors at multiple hand sites (thumb, index finger, and wrist). This multilocation monitoring approach provides more comprehensive than single‐site measurement for accurate tremor detection, both in simulated daily activities (e.g., rest, postural, walking, running, and stair descent).

Also, clinical trials demonstrated that the quantitative tremor indicators (amplitude and frequency) extracted by the system correlated with clinician‐assessed UPDRS III scores and revealed significant differences between PD and ET in some conditions. Specifically, PA exhibited a statistically significant difference in the postural tremor measured at the index location between patients with PD and ET. Furthermore, a statistically significant difference was confirmed between PD rest and ET postural tremors, which represent the characteristics of the two diseases, for measurements obtained at all (average of the thumb, index finger, and wrist) and thumb locations. In addition, in the correlation analysis between TS in UPDRS and tremor indicators, when measurements were conducted at all locations (average of the thumb, index finger, and wrist), tended to be observed higher correlation coefficient and statistical significance than single‐location measurements in many cases. Specifically, this tendency was observed in TS and PA (rest tremor), TS and FF (rest tremor), TS and FF (postural tremor), PA and FF (rest tremor) in PD group and TS and PA (postural tremor) in ET group. These findings demonstrate the potential of our system to act as a wearable platform for objective and quantitative tremor measurement.

## Author Contributions

**Chiwon Song**: conceptualization, data curation, writing – original draft, writing – review and editing, formal analysis, investigation, methodology, validation, visualization, software, project administration. **Chan Park**: conceptualization, investigation, writing – original draft, writing – review and editing, supervision, project administration, methodology. **Juyoun Lee**: writing – original draft, writing – review and editing, conceptualization, formal analysis, investigation, supervision, funding acquisition, resources, project administration, methodology, validation. **Hojoong Kim**: visualization, investigation, validation. **Jeongbeom Kang**: methodology, investigation. **Byeongjun Lee**: investigation, visualization. **Cheoljeong Park**: investigation, software. **Jungmin Kim**: visualization, investigation. **Haran Lee**: investigation. **Yoon Jae Lee**: software, writing – review and editing. **Min‐Kyung Yeo**: supervision, writing – review and editing, conceptualization, resources, project administration, funding acquisition. **Woon‐Hong Yeo**: conceptualization, writing – review and editing, supervision, resources, project administration, funding acquisition. **Seong J. Cho**: writing – original draft, writing – review and editing, supervision, conceptualization, project administration, resources, funding acquisition.

## Funding

This work was supported by the Korea Institute for Advancement of Technology (KIAT) in 2024 (No. RS‐2024‐00435815) and in 2022 (RS‐2022‐KI002563, Human Resource Development Program for Industrial Innovation), by the Institute of Information & Communications Technology Planning & Evaluation (IITP) in 2024 (Nos. RS‐2024‐00422098 and RS‐2024‐00443780), by the National Research Foundation of Korea (NRF) grant (RS‐2026‐25473953), by BK21 FOUR Program by Chungnam National University Research Grant in 2023, 2024 and 2025, by Chungnam National University, by the WISH Center grant from the Georgia Tech Institute for Matter and Systems, by the Global Industrial Technology Cooperation Center (GITCC) through a grant agreement with the Korea Institute for Advancement of Technology (KIAT), and by the National Science Foundation Research Traineeship (Grant No. NRT‐FW‐HTF 2345860).

## Institutional Review Board Statement

This retrospective study was approved by the Chungnam National University Hospital. Institutional board (CNUH IRB 2021‐11‐060), and the authors confirm that written informed consent has been obtained from the involved patients.

## Conflicts of Interest

The authors declare no conflicts of interest.

## Supporting information




**Supporting File**: advs76724‐sup‐0001‐SuppMat.docx.

## Data Availability

The data that support the findings of this study are available from the corresponding author upon reasonable request.
