# Supplementary material for: Highly Repeatable Fibrous Crack‐Based Strain Sensor for Multichannel Continuous Tremor Monitoring in Parkinson's Disease and Essential Tremor
Source: Adv Sci (Weinh). 2026 Aug 2:e76724. Online ahead of print. doi: 10.1002/advs.76724 (PMC13429862; doi:10.1002/advs.76724)
Supplement: Supplementary file 1 — Supporting File: advs76724‐sup‐0001‐SuppMat.docx. [file ADVS-9999-e76724-s001.docx]

Supporting Information

Highly Repeatable Fibrous Crack-based Strain Sensor for Multichannel Continuous Tremor Monitoring in Parkinson’s Disease and Essential Tremor

Chiwon Song, Chan Park, Juyoun Lee, Hojoong Kim, Jeongbeom Kang, Byeongjun Lee, Cheoljeong Park, Jungmin Kim, Haran Lee, Yoon Jae Lee, Min-Kyung Yeo*, Woon-Hong Yeo*, Seong J. Cho*

C. W. Song, J. B. Kang, B. J. Lee, C. J. Park, J. M. Kim, H. R. Lee, S. J. Cho

Department of Mechanical Engineering, Chungnam National University (CNU), Daejeon 34134, Republic of Korea

E-mail: scho@cnu.ac.kr

C. Park

Department of Mechanical Engineering, Chungnam National University (CNU), Daejeon 34134, Republic of Korea

National NanoFab Center (NNFC), Daejeon 34141, Republic of Korea

J. Lee

Department of Neurology, Chungnam National University School of Medicine, Daejeon, 35015 Republic of Korea

M. K. Yeo

Department of Pathology, Chungnam National University School of Medicine, Daejeon, 35015 Republic of Korea

E-mail: mkyeo83@cnu.ac.kr

H. Kim

George W. Woodruff School of Mechanical Engineering, Georgia Institute of Technology,

Atlanta, GA, 30332, USA

Y. J. Lee

Wearable Intelligent Systems and Healthcare Center (WISH Center) at the Institute for Matter and Systems, Georgia Institute of Technology, Atlanta, GA, 30332, USA

School of Electrical and Computer Engineering, Georgia Institute of Technology, Atlanta, GA, USA

Department of Computer Science, Georgia State University, Atlanta, GA, USA

W. H. Yeo

George W. Woodruff School of Mechanical Engineering, Georgia Institute of Technology, Atlanta, GA, 30332, USA

Wearable Intelligent Systems and Healthcare Center (WISH Center) at the Institute for Matter and Systems, Georgia Institute of Technology, Atlanta, GA, 30332, USA

Wallace H. Coulter Department of Biomedical Engineering, Georgia Institute of Technology and Emory University School of Medicine, Atlanta, GA, 30332, USA

Parker H. Petit Institute for Bioengineering and Biosciences, Georgia Institute of Technology, Atlanta, GA, 30332, USA

Korea KIAT-Georgia Tech Semiconductor Electronics Center (K-GTSEC) at the Institute for Matter and Systems, Georgia Institute of Technology, Atlanta, GA, 30332, USA

E-mail: whyeo@gatech.edu

**1. Experimental Section and Methods**

**1.1 Materials**

To fabricate PU fibers, polyurethane beads (Pallethane 2363-80AE; Lubrizol, USA), tetrahydrofuran (99.5 %, Samchun Pure Chemical, Republic of Korea), and N,N-Dimethylformamide (99 %, Samchun Pure Chemical, Republic of Korea) were used. For the fabrication and encapsulation of the sensor substrate, Ecoflex 00-30 (Smooth-On, USA), Ecoflex Gel (Smooth-On, USA), and polystyrene (B17-132-041, LK LABKOREA, Korea) were utilized. To apply silver plating to the fibers, the following chemicals were employed: silver nitrate solution (AgNO3, 0.1M; Yakuri Pure Chemicals, Japan), sodium hydroxide (NaOH, >96.0 %; Samchun Pure Chemical, Republic of Korea), ammonia solution (NH4OH, 28.0 %–30.0 %; Samchun Pure Chemical, Republic of Korea), dextrose (C6H12O6, 99.5 %; Samchun Pure Chemical, Republic of Korea), pure water (7732-18-5, Samchun Pure Chemical, Republic of Korea), and Tin (II) fluoride (99 %, Sigma-Aldrich). For the wiring of the fabricated sensors, silver epoxy (Conductive Epoxy, CW2400; Chemtronics, Seongnam, Gyeonggi, Republic of Korea) was used.

**1.2 Process equipment**

To cure the sensor substrate, a vacuum oven (OV-11, Jeiotech, Republic of Korea) was used. To enhance the ease of plating through surface modification of the PU fiber, plasma treatment was performed using an oxygen plasma system (Cute Plasma System, Femto Science, Korea). For patterning the sensor structure, a laser machine (ML-4040 LASER, Machineshop, Korea and Protolaser U4, LPKF, Korea) was employed. The sensor performance evaluation system consisted of a linear stage (M-112.1DG, Physik Instrumente, Germany), a motor controller (C-863 Mercury Servo Controller, Physik Instrumente, Germany), a source measure unit (B2902A, Keysight, Santa Rosa, CA, USA), and a universal testing machine (UTM, HZ-1007E, MMS TECH, Republic of Korea). For observing the cross-section and surface of the sensors, a field-emission scanning electron microscope (FE-SEM), a scanning electron microscope (EM-30 MiniSEM, COXEM, Korea), and a laser scanning confocal microscope (LSCM, VK-X3000, Keyence, Japan) were used.

**1.3 Characterization Methods**

The surface and cross-sectional morphologies of the sensor were observed using a field-emission scanning electron microscope (FE-SEM), a scanning electron microscope, and a laser scanning confocal microscope (LSCM, VK-X3000, Keyence, Japan). The behavior of fibers and the generation of cracks in the FCBS sensor under strain were monitored using LSCM. The ImageJ software was utilized to measure the length and angle of the fibers captured in the LSCM images. Additionally, the orientation of the fibers in the FCBS sensor under strain was analyzed using the orientation plug-in of ImageJ with the fiber images obtained from LSCM.

**1.4 Preparation of the TPU fiber membrane**

**Figure S2** describes the electrospinning machine and the fabrication conditions for TPU fiber membranes. To fabricate TPU fibers, an 18 wt. % PU solution was prepared by mixing 15 mL of THF, 10 mL of DMF, and 5.4878 g of PU beads in a vial, followed by stirring with a shaker. The prepared PU solution was then loaded into a syringe, which was positioned 16.5 cm away from an aluminum plate. During the electrospinning process, a voltage of 8 kV was applied to the metal tip of the syringe using a high-voltage power supply, while the aluminum plate was grounded. Electrospinning was conducted for 30 min at a flow rate of 1 mL/h. The resulting PU fibers were left to dry at room temperature for 24 h to ensure the removal of any residual solvent. Finally, the PU fibers were carefully detached from the aluminum plate using a PET guide and stored in a petri dish.

**1.5 Fabrication method of Fibrous crack-based strain sensor**

**Figure S3** presents a schematic illustration of the fabrication process for the Fibrous crack-based strain (FCBS) sensor. The detailed fabrication steps are as follows: To prepare the substrate, an Ecoflex mixture was created by combining Ecoflex 00-30 and Ecoflex gel in a 1:1 volume ratio. This mixture was spin-coated onto a polystyrene (PS) substrate at 1000 rpm for 30 s and then cured in a vacuum oven at 60 °C for 30 min. Next, the PU fiber mat was transferred onto the Ecoflex substrate following plasma treatment using oxygen plasma system. The transferred fiber mat was patterned using a laser machine, and a polyethylene terephthalate (PET) film mask was attached to the Ecoflex substrate. The substrate was then immersed in a solution containing 100 mL of pure water and 0.002 g of tin (II) fluoride for 10 min. For silver (Ag) deposition onto the PU fibers, the mat was immersed in Tollens’ reagent solution at 4 min. This solution was prepared by mixing 30 mL of silver nitrate, 3 mL of sodium hydroxide, 1.05 mL of ammonia solution, and 0.27 g of dextrose. To enable electrical signal measurement, the Ag-coated fibers were connected using copper wires and conductive silver epoxy. The assembly was then cured at 60 °C for 30 min. Finally, the substrate was encapsulated by spin-coating with the Ecoflex mixture at 1000 rpm for 30 s, followed by baking in an oven at 60 °C for 30 min.

**1.6 Human subject study and Tremor data signal processing**

The clinical study was conducted in compliance with ethical research guidelines approved by the Chungnam National University Hospital Institutional Board (CNUH IRB 2021-11-060). Patients participating in the study provided written informed consent before the start of the study. **Figure S19** presents tremor data signal processing processes. Hand tremor data were collected from 11 Parkinson's disease (PD) patients, 10 Essential Tremor (ET) patients using a Hand Tremor Monitoring System. Each patient was instructed to assume two postures while seated in a chair. First, the patient remained relaxed with the arm supported on the armrests. Second, the patient extended their arms to the forward and maintained the posture against gravity. Immediately after assuming the instructed postures, the patients performed maximum flexion and extension movements of the thumb, index finger, and wrist, and were then instructed to maintain the given posture. Subsequently, voltage variations caused by tremors in the thumb, index finger, and wrist were measured using a flexible circuit at a sampling rate of 500 Hz. The collected data were transmitted to a smartphone via Bluetooth. For each patient, tremor data from the thumb, index finger, and wrist, collected in various postures, were normalized based on the voltage difference between the maximum flexion and extension states. To eliminate non-tremor signals, a 5th-order Butterworth filter was applied for band-pass filtering in the frequency range of 1 Hz to 16 Hz. The filtered data were segmented into 1-second intervals with a 50 % overlap, and a Hamming window was applied to each segment. These segmented data were processed using a Fourier Transform to calculate the Power Spectrum Density (PSD). The following signal features were extracted from each segment:

Peak Amplitude (PA): The maximum value of the PSD

Fundamental Frequency (FF): The frequency at which the PSD reaches its maximum value
Segments with FF values within the range of 3 Hz to 12 Hz were selected, and the top 10 % of these segments based on PA values were identified. Finally, the features of these selected segments were averaged for each patient and recorded alongside the tremor scores (tremor score) assessed by the physician for each patient.

All locations, which are the averaged representative values ​​of single locations, were used to reduce variability caused by location-dependent systematic error and random error.

Expressed mathematically, the signal measured at a measurement location of patients can be estimated by the following equation:

$$y_{p}^{(l)}=s_{p}+b_{p}^{(l)}+{}_{p}^{(l)} (1)$$

In Equation (1), $y_{p}^{(l)}$is measured tremor signal, $s_{p}$ is the true tremor signal, and $b_{p}^{(l)}$ and ${}_{p}^{(l)}$ are respectively the location-dependent systematic error and random error, measured at the patient ($p$) measurement location ($l$).

According to Equation (1), if only a specific single location is considered, $y_{p}^{(l)}$ may be substantially affected not only by the random error ${}_{p}^{(l)}$ and location-dependent systematic error $b_{p}^{(l)}$. Consequently, the variability in tremor generation across the measurement location and random error can potentially influence the measured tremor signal value and alter rank order of $y_{p}^{(l)}$, thereby directly affecting rank-based analyses. If all measurement locations are considered, the measured tremor signal $y_{p}^{(avg)}$ considering all measurement locations simply expressed as follows:

$$y_{p}^{(avg)}=\frac{1}{L}\sum_{l} y_{p}^{(l)}=s_{p}+\bar{b}_{p}+\bar{}_{p}=s_{p}+\bar{e}_{p} (2)$$

In Equation (2), L is defined as the number of measurement locations, and $\bar{e}_{p}$ is defined as the averaged total error including $\bar{b}_{p}$ and $\bar{}_{p}$. According to Equation (2), the average of the measurement tremor signal $y_{p}^{(avg)}$ may mitigate the variance of location-dependent systematic error and random error, and consequently, the variability between patients may be mitigated compared with single location conditions. Specifically, the variance of total error may be mitigated through averaging, depending on the covariance as expressed in Equation (3).

$$Var{(\bar{e}}_{p})=\frac{1}{L^{2}}\left[ \sum_{l=1}^{L} Var{(e}_{p}^{(l)})+2\sum_{m<l} Cov(e_{p}^{\left( m \right)},e_{p}^{(l)}) \right] (3)$$

In Equation (3), m is defined as another measurement location index different from $l$. For Example, If tremor signals are measured at three locations (thumb, index, wrist), $L=3$, and location indices $(l)$ assigned as thumb = 1, index = 2, wrist = 3, According to above assumption, covariance term $m<l$ assigned pairs (thumb, index), (thumb, wrist), (index, wrist).

**1.7 Sensor evaluation system**

The evaluation setup for the sensor is detailed as follows. To apply strain to the sensor, it was mounted on a linear stage controlled by a motor controller. The current changes due to sensor deformation were measured using a Source Measure Unit (SMU). LabVIEW software was utilized to control the linear stage, record displacement, resistance data from the SMU at defined time intervals, enabling precise monitoring of both sensor deformation and corresponding resistance variations. The measurement system for determining mechanical properties comprised a Universal Testing Machine (UTM), capable of measuring both displacement and force applied to the sensor, and custom software for controlling the UTM and logging data. To measure Young's modulus, a portion of the sensor was fixed on the UTM’s hydraulic and mechanical jigs. Subsequently, a strain rate of 1 % per second was applied, during which time, force, and displacement data were collected.

**1.8 PSD ratio of involuntary to voluntary movement (PRIV) calculation**

The Power spectral density is estimated by classical periodogram method using python (Equation S1).

$PSD\left( f \right)=\frac{{\mid X\left( f \right)\mid}^{2}}{n*f_{s}} \left( S1 \right)$

In Equation (S1), Parameters demonstrate that X(f): result of Fast Fourier Transform (FFT) applied to the voltage signal in the time domain, n: Amount of data in the segment, fs: sampling frequency.

Based on the result of equation S1, we calculated the PSD ratio of involuntary to voluntary movement (PRIV) for determine which location is more effective for tremor detection under behavior.
The PRIV was calculated (Equation (S2)) as the ratio between the PSD in the tremor frequency range (8–10 Hz) and the PSD in the low-frequency range (0.5–3 Hz) associated with various movements. (b) and location (l; thumb, index, wrist). Max normalization was then performed using the location with the highest PRIV for each behavior (Equation (S3)).

$\frac{Involuntary movement}{Voluntary movement}PSD ratio={PSD}_{b}=\frac{\sum_{8Hz}^{10Hz} PSD\left( f \right)}{\sum_{0.5Hz}^{3Hz} PSD\left( f \right)} for b\in\{Rest, Postural, Shoulder shaking,Walking, Running, Up stair, Down stair\}$ $(S2)$

$$Normalized\frac{Involuntary movement}{Voluntary movement}PSD ratio=\tilde{PSD}_{b}^{\left( l \right)}$$

$=\frac{\tilde{PSD}_{b}^{\left( l \right)}}{Max\left( \tilde{PSD}_{b}^{Thumb},\tilde{PSD}_{b}^{Index},\tilde{PSD}_{b}^{Wrist} \right)} for l\in\left\{ thumb,index,wrist \right\}$ $(S3)$

**2. Composite structural design for FCBS sensor**

In this section, we specifically demonstrate to composite structural design and mechanism for enhancing performance of FCBS sensor. CBS are suitable as wearable tremor sensors for tremor monitoring due to their high sensitivity to micro-vibrations and excellent mechanical flexibility.^[1, 2]^ Many studies have focused on thin-film crack-based strain (TCBS) sensors, but, cracks localized to 2D conductive layer limit working range and repeatability **(Figure 2b)**.^[3-6]^ On the other hand, the FCBS sensor provides stable and reliable signals over a wide working range, because cracks are generated and propagated differently in each fiber strand within 3D fiber network composed of randomly oriented PU fibers formed through electrospinning **(Figure 2c)**. Also, we applied composite structural design including a crack structure, random fiber orientation, junction melting and polymer penetration within a 3D fiber network to FCBS sensor **(Figure S6)**. The electrospinning equipment and fabrication process used for fabrication of FCBS sensors are detailed in **Figure S2 and S3**. As a result, The FCBS sensor achieves a working range ten times wider than TCBS sensor, superior repeatability of over 100,000 cycles and skin-like modulus of 45.46 kPa **(Figure 2d, f–g)**, suggesting that the FCBS sensor is suitable as a wearable sensor for tremor in continuous monitoring. In the following paragraphs describe in detail about to each composite structural design (crack structure, random fiber orientation, junction melting, polymer penetration) effect of FCBS sensor’s performance.

**2.1 Random fiber orientation and crack structure**

The random orientation of fiber formed by the electrospinning process contributes to the wide working range of the FCBS sensor by maintaining electrical connectivity under large strain, resulting in a ten-times wider working range than TCBS sensor **(Figure 2d)**. This performance improvement originates from the variation of crack generation and propagation that occur differently for each fiber strand under strain, crack generation and propagation is determined by the orientation angle of each fiber relative to the strain direction **(Figure S6a)**.^[7, 8]^ **Figure S7**, **Table S3** shows the reorientation process of randomly oriented fibers under increasing strain. Initially, the three-dimensional fiber network formed by electrospinning has a random orientation but is gradually reoriented along with the tensile direction. Specifically, at strain conditions of 0%, 30%, 60%, 90%, 120%, 150%, and 180%, the average fiber orientation angle (Gaussian fitting center) decreases progressively to 13.18°, 9.81°, 5.62°, 1.85°, 0.29°, −0.20°, and −0.24°, respectively. During the reorientation process of randomly oriented fibers, fibers with smaller initial angles that are defined as angles between each fiber and the tensile direction at 0% strain, tend to rapidly increase crack length to total fiber length ratio **(Figure S8, Table S4, S5)**. Specifically, four fibers with initial angles of –6.32°, 28.73°, 51.96°, and –84.52° recorded crack ratios of 63.76%, 60.15%, 41.82%, and 24.74%, respectively, at 180% strain. **Figure S8** visually and quantitatively shows the change in the crack length-to-total fiber length ratio according to strain for each initial fiber angle case. These results suggest that the initial orientation of fibers controls crack generation and propagation under strain. Accordingly, random cracks associated with randomly oriented fibers contribute to the wide working range of the FCBS sensors by maintaining continuous conductive pathways under strain (**Figure 2d**).

**2.2 Junction melting**

The FCBS sensor exhibits stable electrical response under 100,000 repeated tensile cycles **(Figure 2g)**. The high signal stability of FCBS sensor is induced by junction melting, which refers to fiber fusion induced by residual organic solvent at fiber junction. This junction melting enhances the mechanical bonding between fibers, thereby improving the structural stability of fibers and contributing to sensor’s electrical signal stability **(Figure S6b)**.^[9-11]^ Junction melting was controlled by adjusting the tip-to-collector distance (TTCD).^[12-14]^ **Figure S9** visually illustrates fibers that controlled junctions melting according to the TTCD. At short TTCD (9.5, 16.5cm) a higher residual organic solvent remained in the fiber membrane, we can observe many junctions melting between fibers **(Figure S9a, b)**. In contrast, at long TTCD (25cm, 30cm), junction melting was not observed due to sufficient evaporation of organic solvent during the electrospinning process **(Figure S9c, d)**. However, an excessively short TTCD (e.g., 9.5cm) resulted in a fiber membrane diameter of less than 5cm. This small diameter caused excessive organic solvents to remain in the fiber membrane, leading to fiber network collapse and the formation of a flat film in the central region of fiber membrane. Therefore, we fabricated the FCBS sensor using the fiber membrane produced under 16.5cm TTCD that can form appropriate fiber membrane diameter (12 cm) for sensor fabrication and stably junction melting. The fabricated FCBS sensor showed excellent signal stability compared to the TCBS sensor in the 1,000 cycle tensile test results shown in **Figure S12**. Additionally, in repeat tensile test conducted over 100,000 cycles at 10% strain, the FCBS sensor with sufficient junction melting **(Figure 2h red)** maintained 80.73% of its initial sensitivity (GF: 7.27 → 5.87), while the FCBS sensor with suppressed junction melting **(Figure 2h blue)** showed a sensitivity reduction rate of 82.08% (GF: 6.64 → 1.19) compared to the initial sensitivity. As a result, the optimized FCBS sensor showed approximately 4.5 times higher sensitivity stability than the sensor with suppressed junction melting. These results suggest that the mechanical structural stability improvement achieved through fiber bonding via junction melting significantly improves the stability of electrical signals under repeated tension. Such electrical signal stability supports the suitability of the FCBS sensor for tremor monitoring applications that require repeated and rapid signal measurement, highlighting its potential for clinical applicability.

**2.3 Polymer penetration in porous structure**

The porous structure formed by the 3D fiber network enables polymer penetration during the packaging process **(Figure S6c)**, significantly improving the sensor's stability (100,000 cycles) and sensitivity (GF: 17.93 under 60%, 57.53 at 60–140%, 476.89 at 140–200%), and Young's modulus (45.46 kPa) **(Figure 2d, f, h)**.^[15-18]^ **Figure S6c** visually illustrates the cross-section of the packaged FCBS sensor. During the packaging process, the liquid-state Ecoflex polymer penetrates the 3D fiber network, effectively filling the voids between fibers and solidifying. This strengthens the bond between the Ecoflex substrate and the fibers, preventing fiber sliding or delamination of the conductive layer deposited on the fiber surfaces, thereby contributing to the stable maintenance of the sensor's electrical signals even under repeated tensile strain exceeding 100,000 cycles **(Figure 2h red)**.^[15, 16]^ Additionally, Ecoflex, which fills the porous structure of the fiber, has a low Young’s modulus, contributing to the excellent mechanical flexibility of the FCBS sensor.^[17, 18]^ As a result, the FCBS sensor exhibits a low Young's modulus of 45.46 kPa, which is approximately 15.57% lower than that of the TCBS sensor with the same thickness of TPU layer, providing mechanical properties like skin **(Figure 2f)**. Also, polymer penetration prevents fiber sliding that is generated fiber sliding between the Ag plated fiber strands during sensor’s deformation, contributes to improving the sensor's sensitivity inducing a sharp increase in resistance. As a result, the packaged FCBS sensor (GF: 476.89 at 140–200%) showed a maximum 59.84% improvement in sensitivity compared to the unpackaged FCBS sensor (GF: 298.39 at 140–200%) **(Figure 2d red, purple)**. These results suggest that penetration of polymers into the fiber network through the packaging process improves the sensitivity, signal stability, and good flexibility of the FCBS sensor, thereby enhancing its applicability as a wearable sensor that can be directly worn on the skin to detect tremors continuously.
 The FCBS sensor we developed is a flexible wearable sensor optimized for tremor measurement, satisfying the required high sensitivity (maximum GF: 476.89), wide working range (maximum working range: 200% strain), excellent repeatability (100,000 cycles), and low Young's modulus (45.46 kPa) similar with skin. This outstanding performance is achieved through the following composite structural design. First, the 3D conductive fiber network based on crack structures and random fiber orientation gradually reorients fiber direction according to tensile strain, forming fibers with varying crack generation and propagation at each fiber that have different orientation angles. thereby contributing to a 10 times wider working range compared to the TCBS sensor. Second, junction melting caused by residual organic solvents at fiber junction strengthens the mechanical bonding between fibers, contributing to excellent repeatability (100,000 cycles) by improving structural, electrical signal stability. Third, polymer penetration during the packaging process fills the internal space of the porous fiber network, suppressing fiber sliding, conductive layer delamination, and recontact between conductive fibers, thereby contributing to improved sensor sensitivity, signal stability, and mechanical flexibility (45.46 kPa). The optimization of this complex structural design suggests that the FCBS sensor has high reliability and applicability as a bio-measurement wearable sensor that can stably measure tremors that occur repeatedly when attached to actual skin.

**
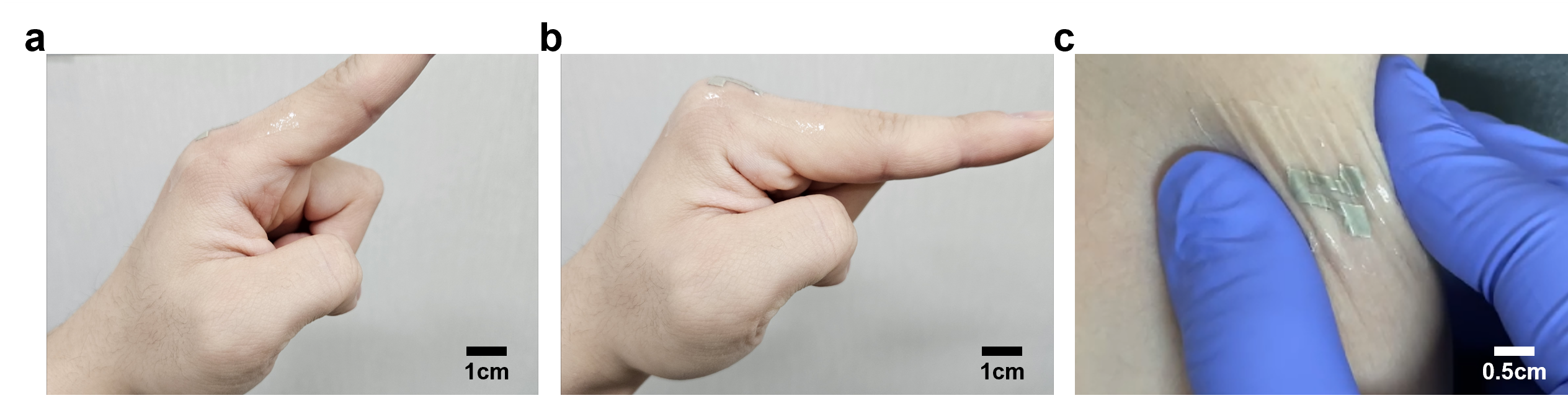
**

**Figure S1.** Self-adhesion test in (a) Extended finger (b) Bent finger skin compliance test in (c) forearm.

**Table S1**. Materials prices of Silver nitrate, MXene, Carbon nanotube, Graphene

| Material | Material price  [$/g] |
| --- | --- |
| Silver nitrate | 6.02 |
| Titanium Carbide MXene | 287 |
| Carbon nanotube single-walled | 112.2 |
| Graphene | 878 |

All material prices were obtained from sigma-Aldrich (https://www.sigmaaldrich.com/) as of April 2026

*FCBS sensor using silver formed from silver nitrate as precursor

**Table S2.** Raw material price of FCBS sensor.

| Material | Material price  [$/g, mL] | Material consumption [g, mL/sensor] | Sensor price  [$/sensor] |
| --- | --- | --- | --- |
| Ecoflex 00-30 | 0.043 | 0.556 | 0.149 |
| Ecoflex Gel | 0.046 | 0.556 |  |
| Tetrahydrofuran | 0.015 | 0.014 |  |
| Dimethylformamide | 0.005 | 0.009 |  |
| Thermoplastic polyurethane | 0.017 | 0.005 |  |
| Silver(I) nitrate | 0.056 | 1.667 |  |
| Sodium hydroxide | 0.007 | 0.167 |  |
| Ammonia | 0.006 | 0.058 |  |
| pure water | 0.001 | 6.389 |  |
| Dextrose | 0.007 | 0.015 |  |
| Tin(II) fluoride | 1.451 | 0.0001 |  |


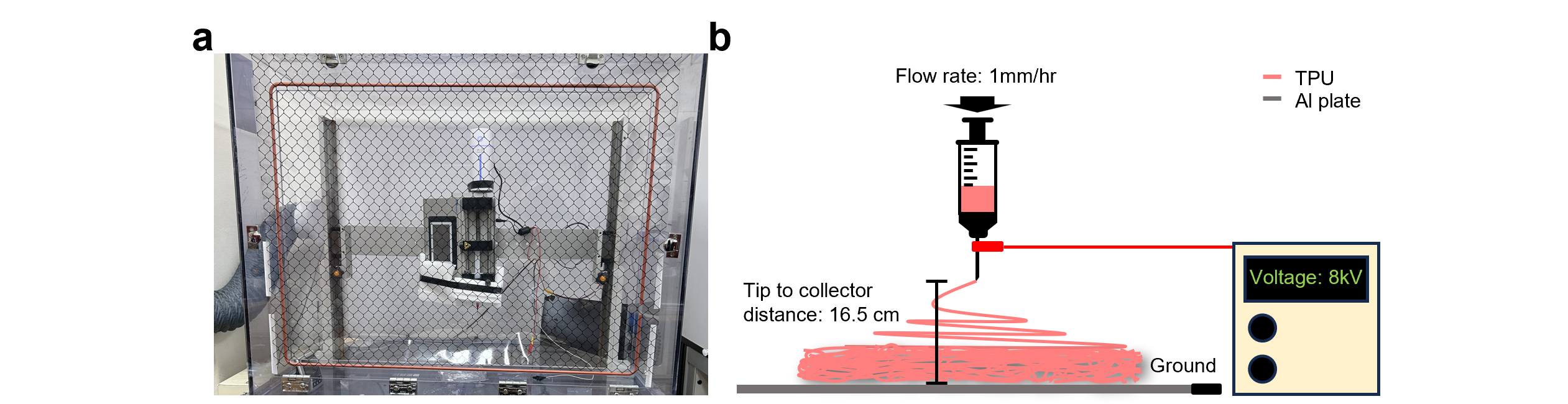


**Figure S2.** image of (a) Electrospinning machine, (b) TPU fiber mat fabrication method.


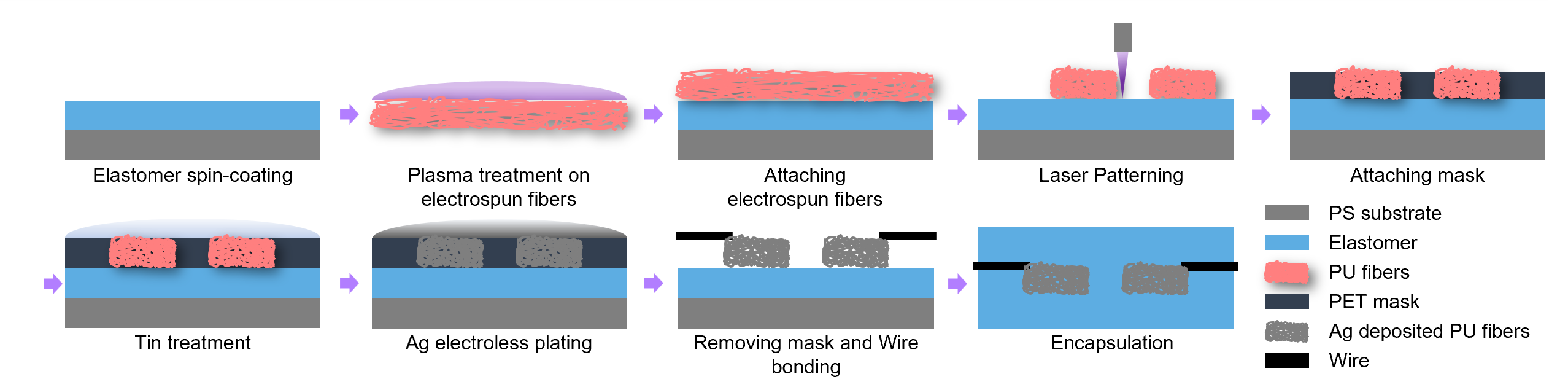


**Figure S3.** Fabrication method of fibrous crack-based strain sensors.


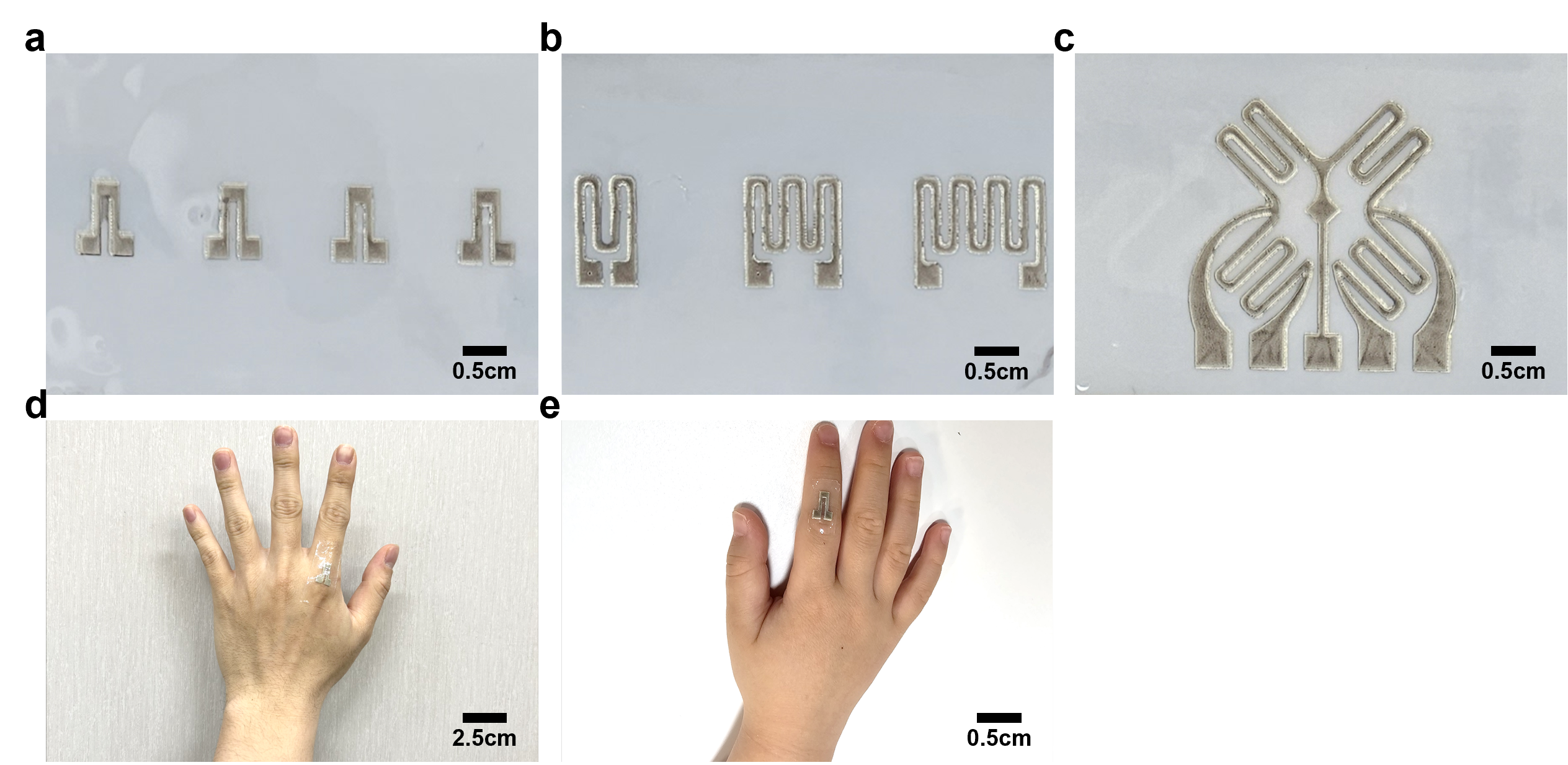


**Figure S4.** Fibrous crack-based strain sensors that have various sizes and shapes
(a) Small size version, (b) Linear strain gauge shape, (c) Full bridge shape. Optimized size to (d) Male hand, (e) Female hand.


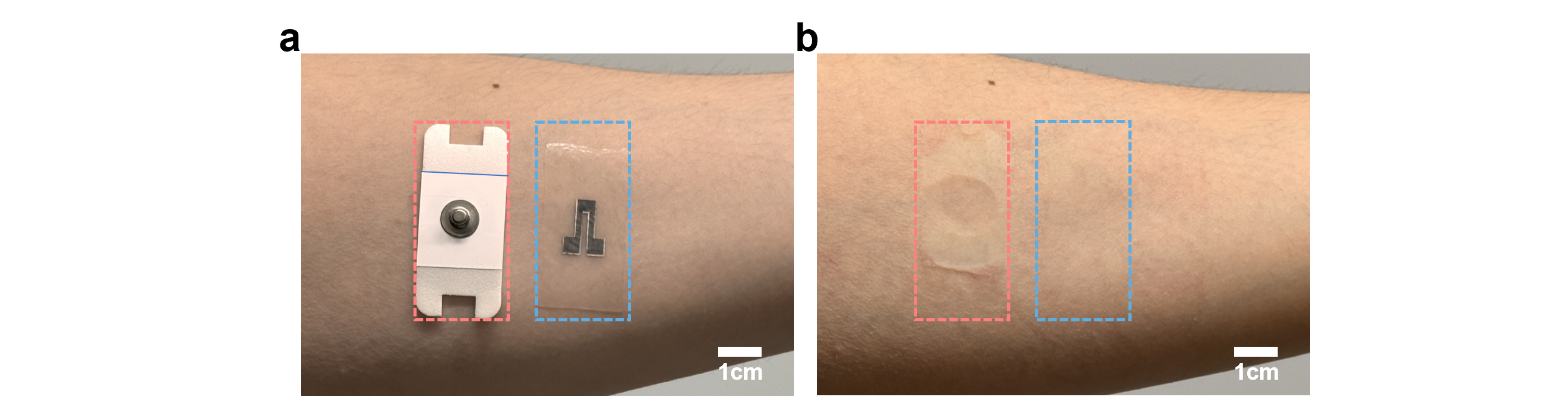


**Figure S5.** 24–hour wearing test of commercial gel electrode and FCBS sensor. Participants performed daily routine such as eating, walking, office work and sleeping. The experiment was conducted under 8–22 °C ambient temperature range. (Daejeon, Republic of Korea)
(a) Commercial gel electrode (left) and FCBS sensor (right) attached to the forearm.

(b) Skin condition after 24 hours later (Left: commercial gel electrode, Right: FCBS sensor).


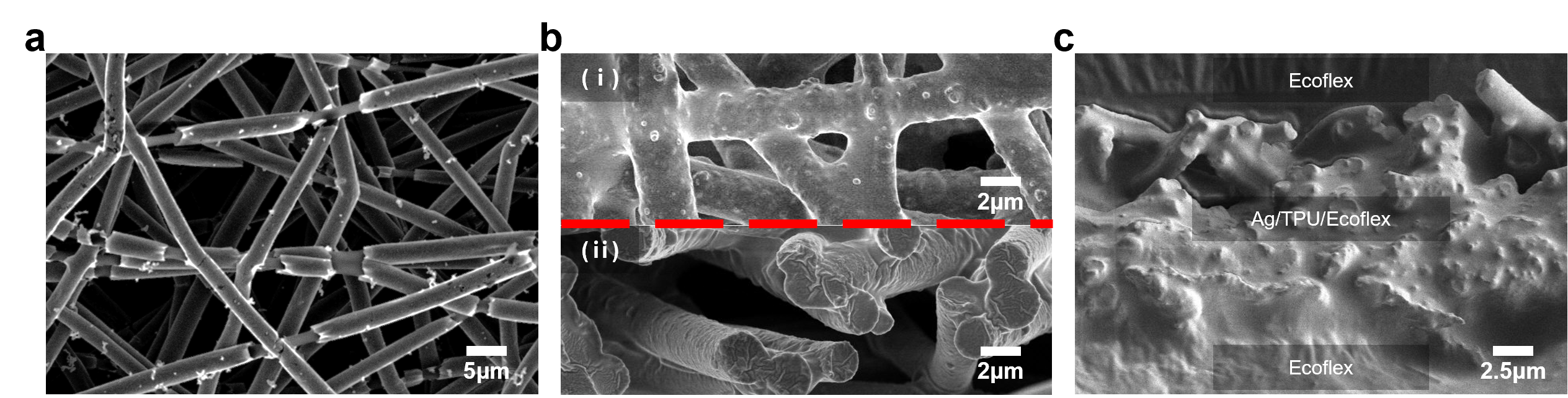


**Figure S6.** (a) SEM image of crack on random orientation fiber (b) SEM image of welded junction Ag/TPU fiber that have melted junction (I) Top and (II)Side view. (c) SEM image showing the cross-section of the FCBS sensor.


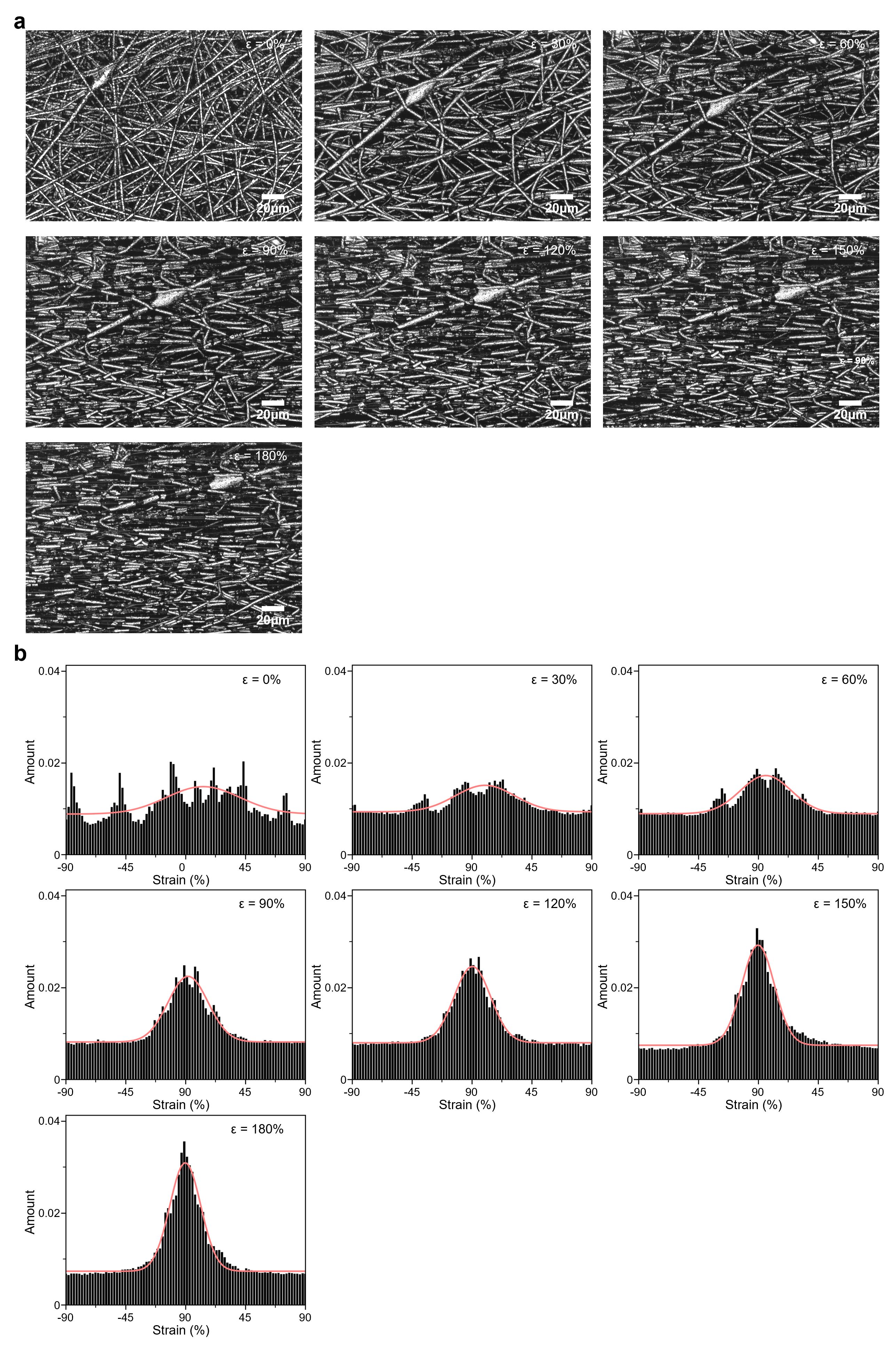


**Figure S7.** (a) Laser microscopy images of Ag/TPU fiber according to strain. (b) Directionality histogram and gaussian fitting curve of Ag/PU fiber according to strain.

**Table S3.** Center angle and standard deviation of Ag/PU fibers according to strain.

| Indicator | Strain  [%] | | | | | | |
| --- | --- | --- | --- | --- | --- | --- | --- |
|  | 0% | 30% | 60% | 90% | 120% | 150% | 180% |
| Center angle [deg] | 13.18 | 9.81 | 5.62 | 1.85 | 0.29 | -0.20 | -0.24 |
| Standard Deviation [deg] | 27.68 | 22.25 | 19.07 | 14.99 | 13.67 | 12.20 | 11.61 |


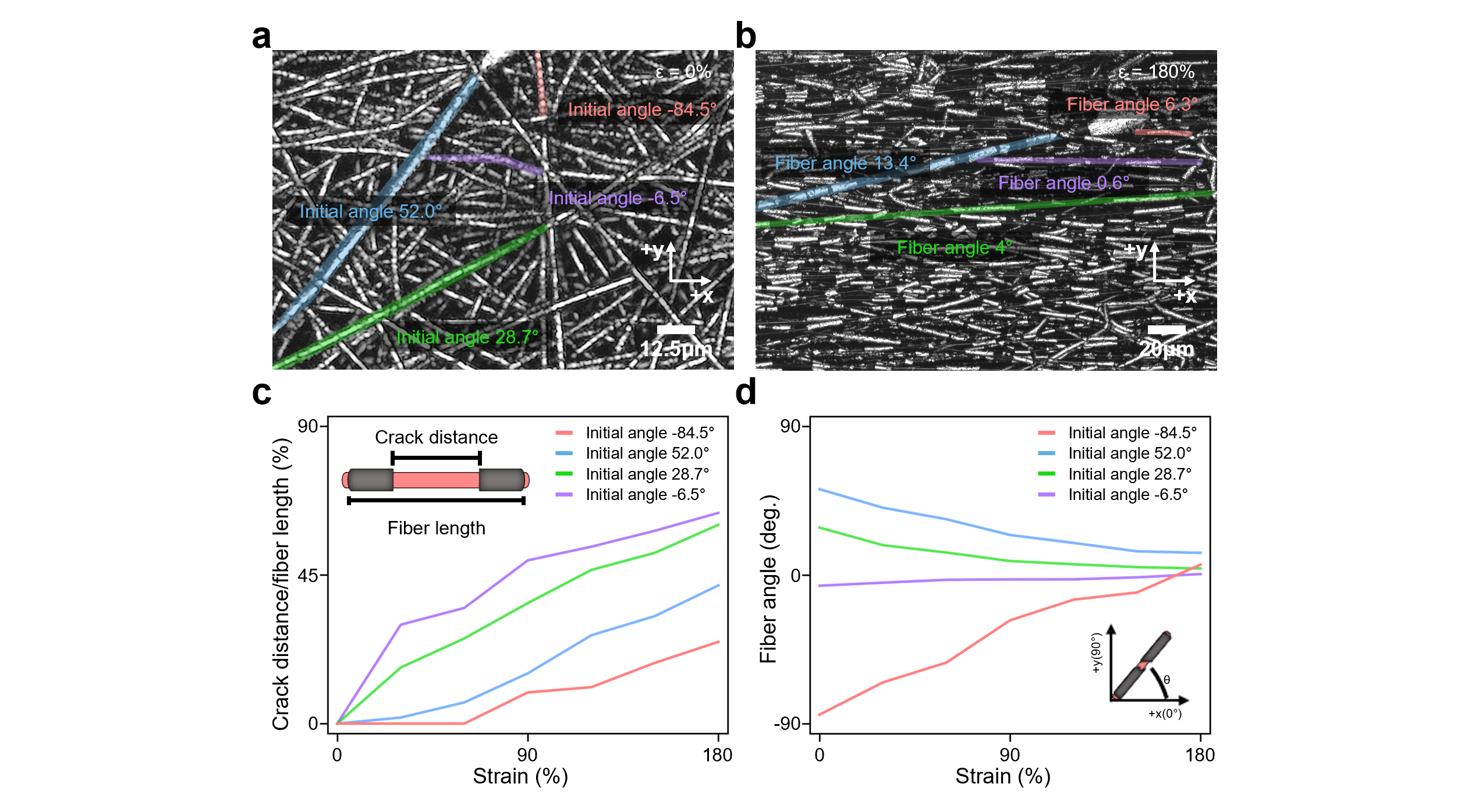


**Figure S8.** Ag/PU fiber behavior according to strain using laser image (a) at 0% strain (initial fiber angle: green(-84.5°),red(52.0°),blue(28.7°),orange(-6.5°) (b) at 180% strain (fiber angle: green(6.32°),red(13.43°),blue(3.97°),orange(0.60°) (c) Fiber angle-strain graph, (d) crack length ratio – strain graph.

**Table S4.** Quantitative data table of fiber angle according to strain.

| Fiber Number | Strain  [%] | | | | | | |
| --- | --- | --- | --- | --- | --- | --- | --- |
|  | 0% | 30% | 60% | 90% | 120% | 150% | 180% |
| Fiber 1 | -6.47 | -4.46 | -2.29 | -2.64 | -2.60 | -1.40 | 0.60 |
| Fiber 2 | 28.73 | 18.06 | 13.61 | 8.48 | 6.52 | 4.78 | 3.97 |
| Fiber 3 | 51.96 | 40.74 | 33.76 | 24.25 | 19.43 | 14.35 | 13.43 |
| Fiber 4 | -84.52 | -64.96 | -52.95 | -27.42 | -14.91 | -10.54 | 6.32 |

**Table S5.** Quantitative data table of crack length / total fiber length (%).

| Fiber Number | Strain  [%] | | | | | | |
| --- | --- | --- | --- | --- | --- | --- | --- |
|  | 0% | 30% | 60% | 90% | 120% | 150% | 180% |
| Fiber 1 | 0 | 29.92 | 35.01 | 49.40 | 53.53 | 58.35 | 63.76 |
| Fiber 2 | 0 | 16.98 | 25.77 | 36.46 | 46.5 | 51.68 | 60.15 |
| Fiber 3 | 0 | 1.82 | 6.41 | 15.21 | 26.72 | 32.53 | 41.82 |
| Fiber 4 | 0 | 0 | 0 | 9.43 | 11.02 | 18.37 | 24.74 |


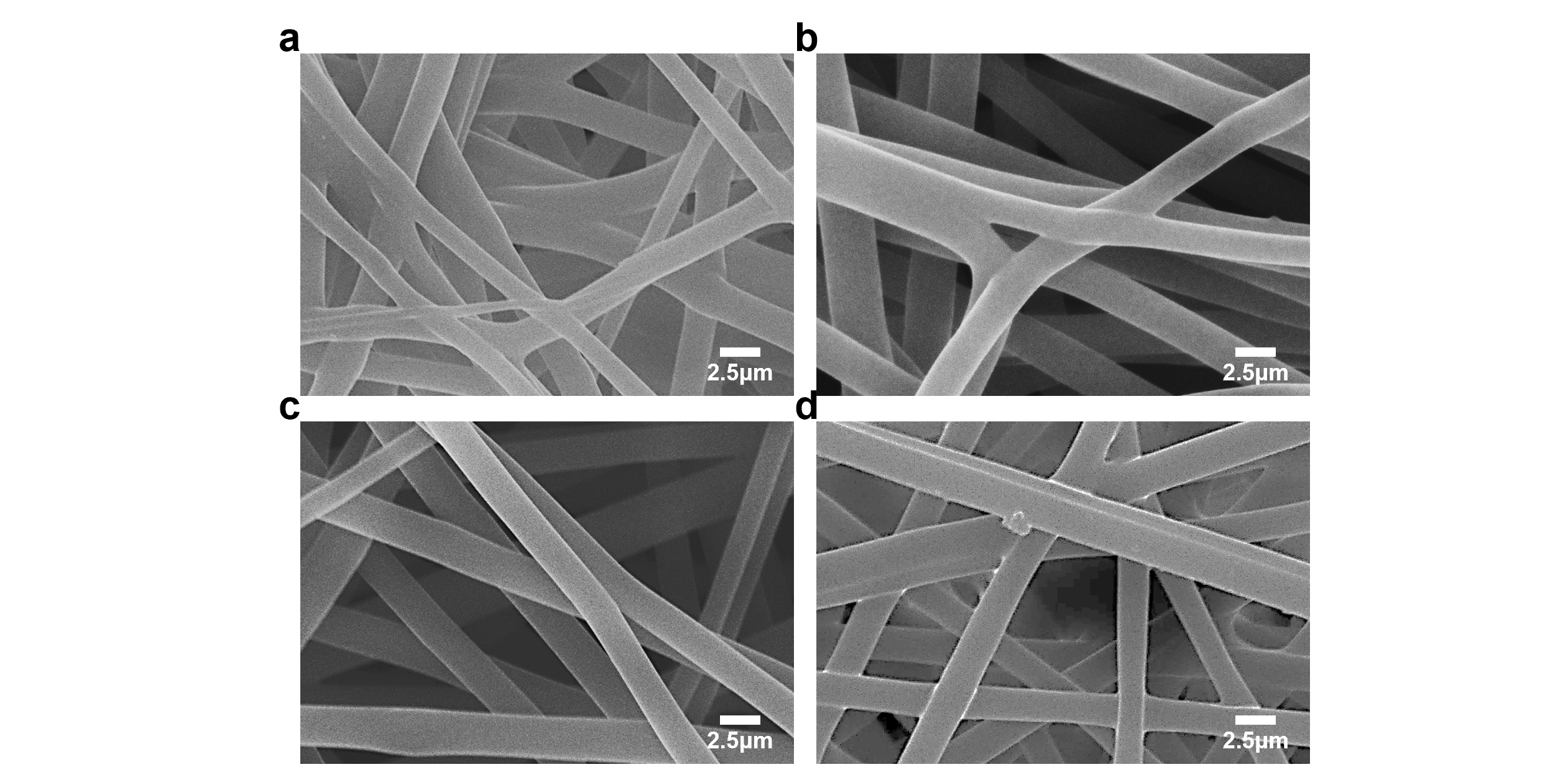


**Figure S9.** SEM image of TPU fiber network according to tip distance (a) 9.5cm, 8kV (b) 16.5cm, 8kV (c) 25cm, 8kV (d) 30cm 14kV.


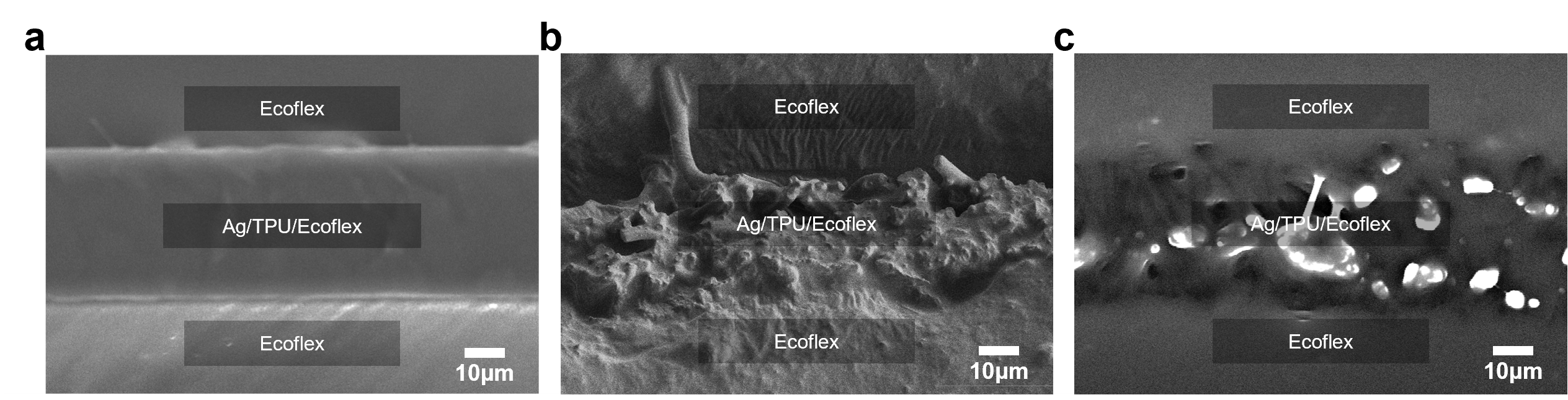


**Figure S10.** SEM Image: cross section of (a) TCBS sensor, (b) FCBS sensor (8kV, Tip distance: 16.5cm) (c) FCBS sensor (14kV, Tip distance: 30cm).


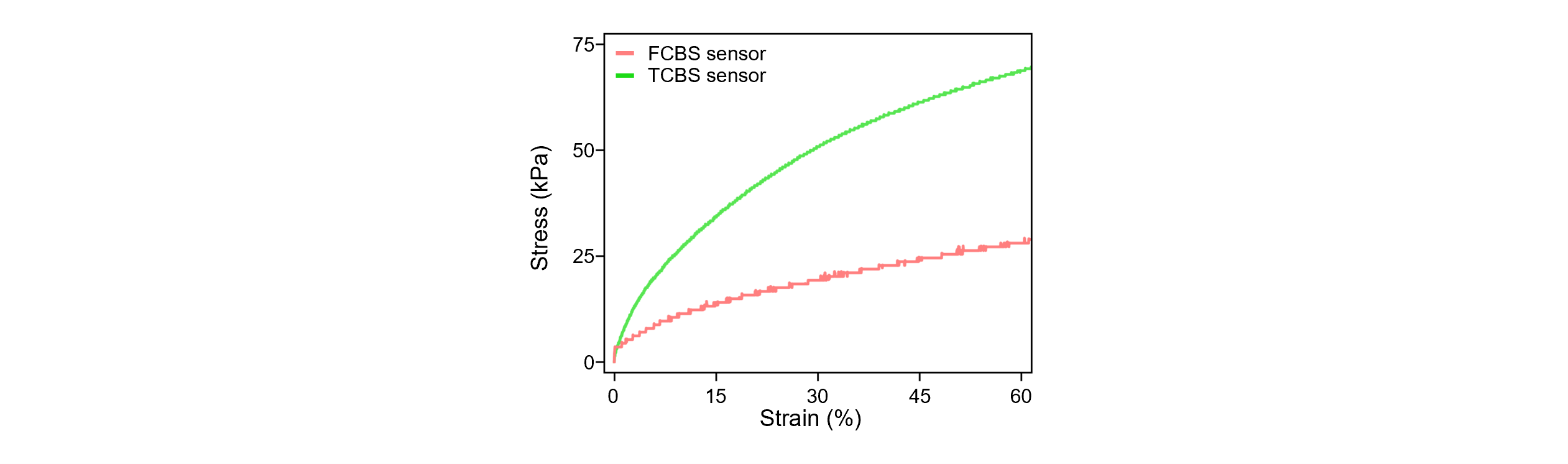


**Figure S11.** Strain-stress curve of TCBS and FCBS sensor at 0 to 60% strain.


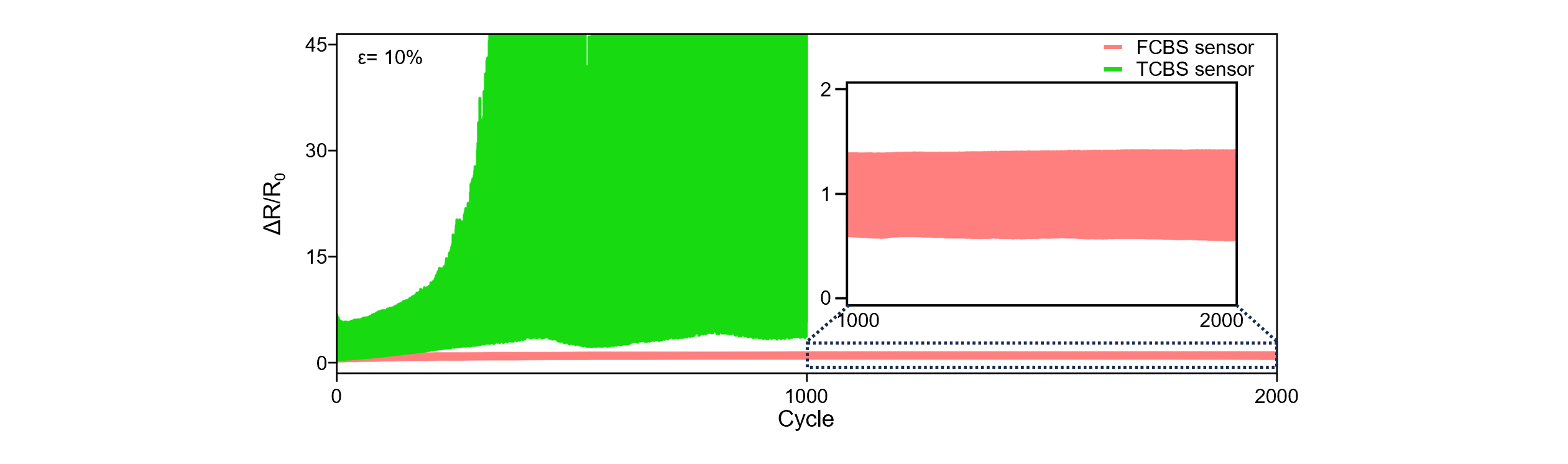


**Figure S12.** FCBS sensor and TCBS sensor tensile test.


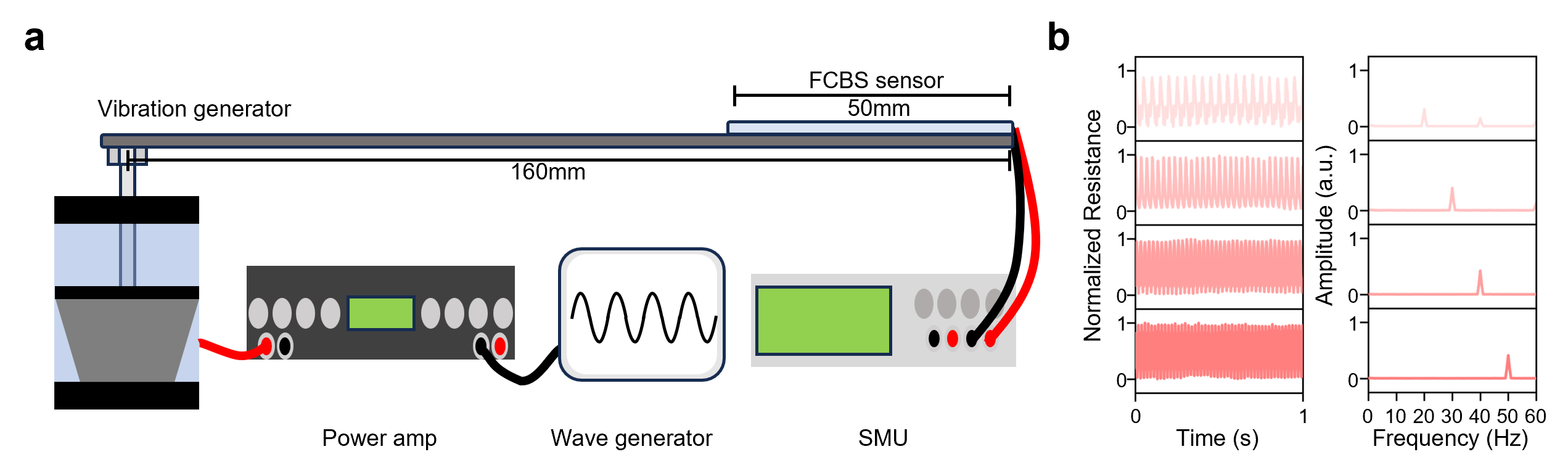


**Figure S13.** Frequency measurement using vibration generation system (a) schematic of vibration generation system. Frequency measurement result at (b) 20–50 Hz


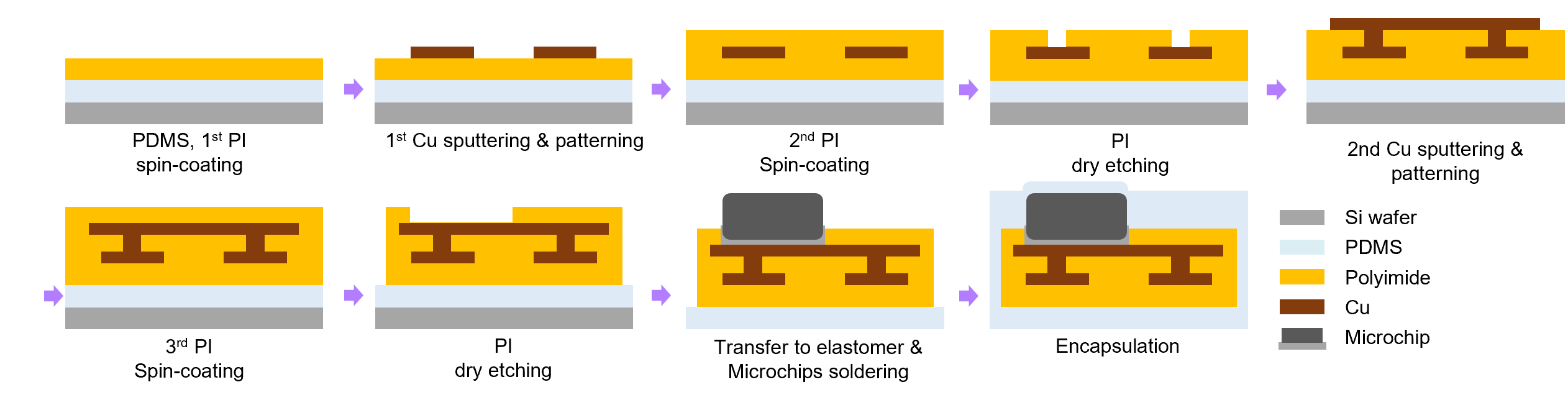


**Figure S14.** Fabrication method of flexible circuits.


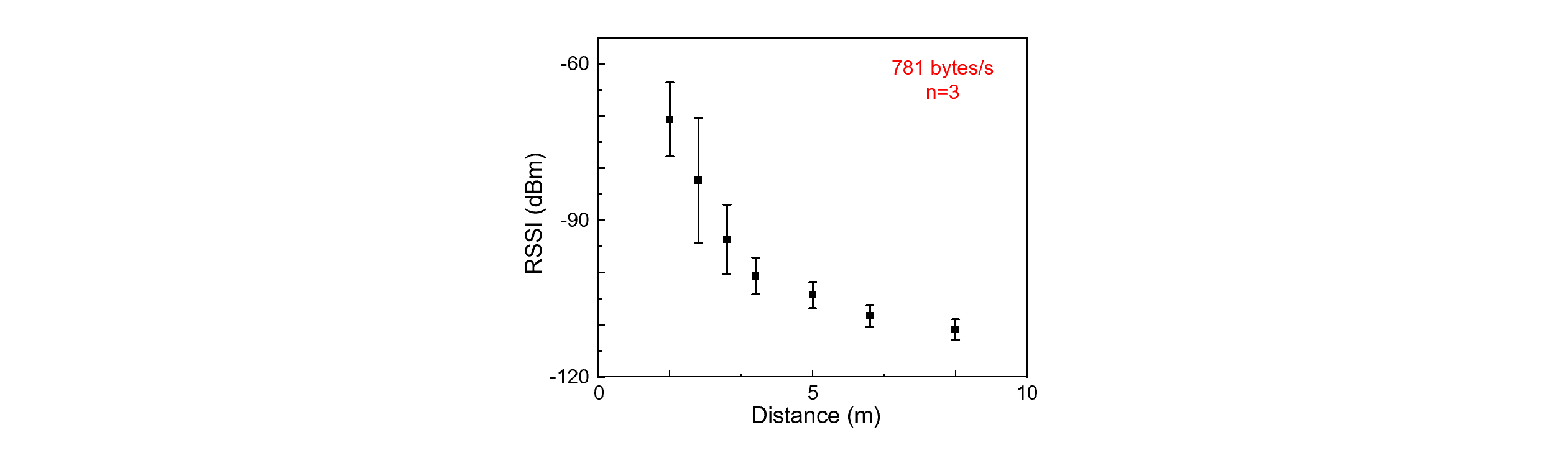


**Figure S15.** RSSI of flexible circuit with smartphone.


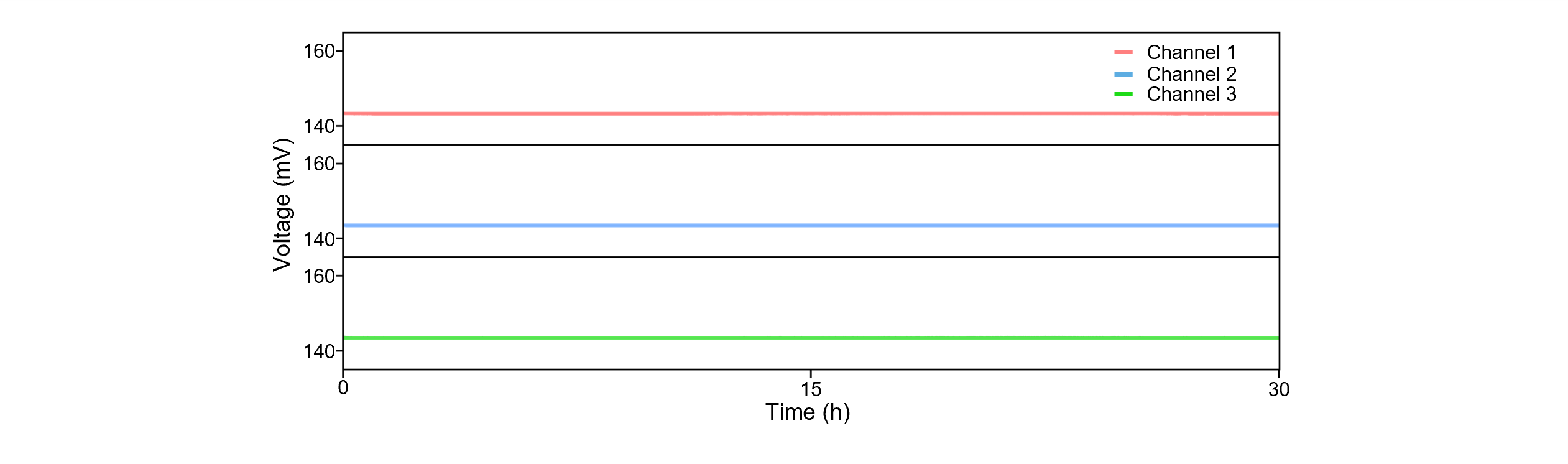


**Figure S16.** Battery lifetime test (600mAh).


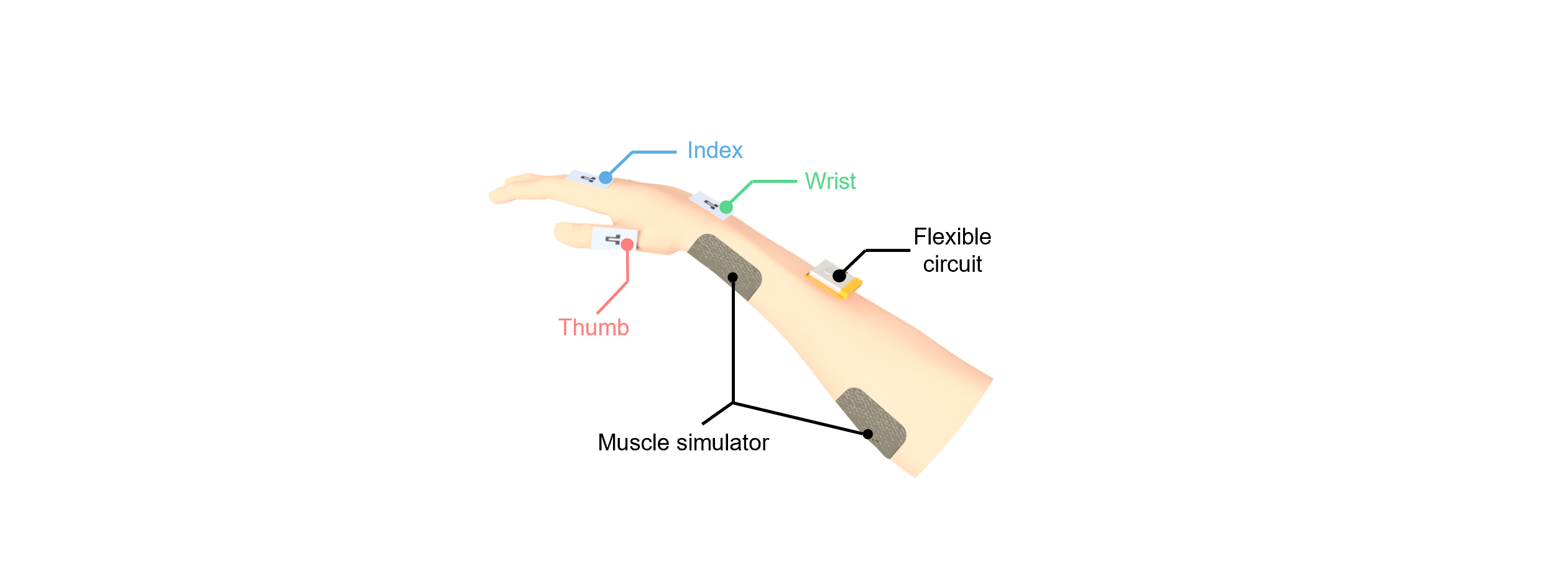


**Figure S17.** Schematic of tremor measurement test setup.


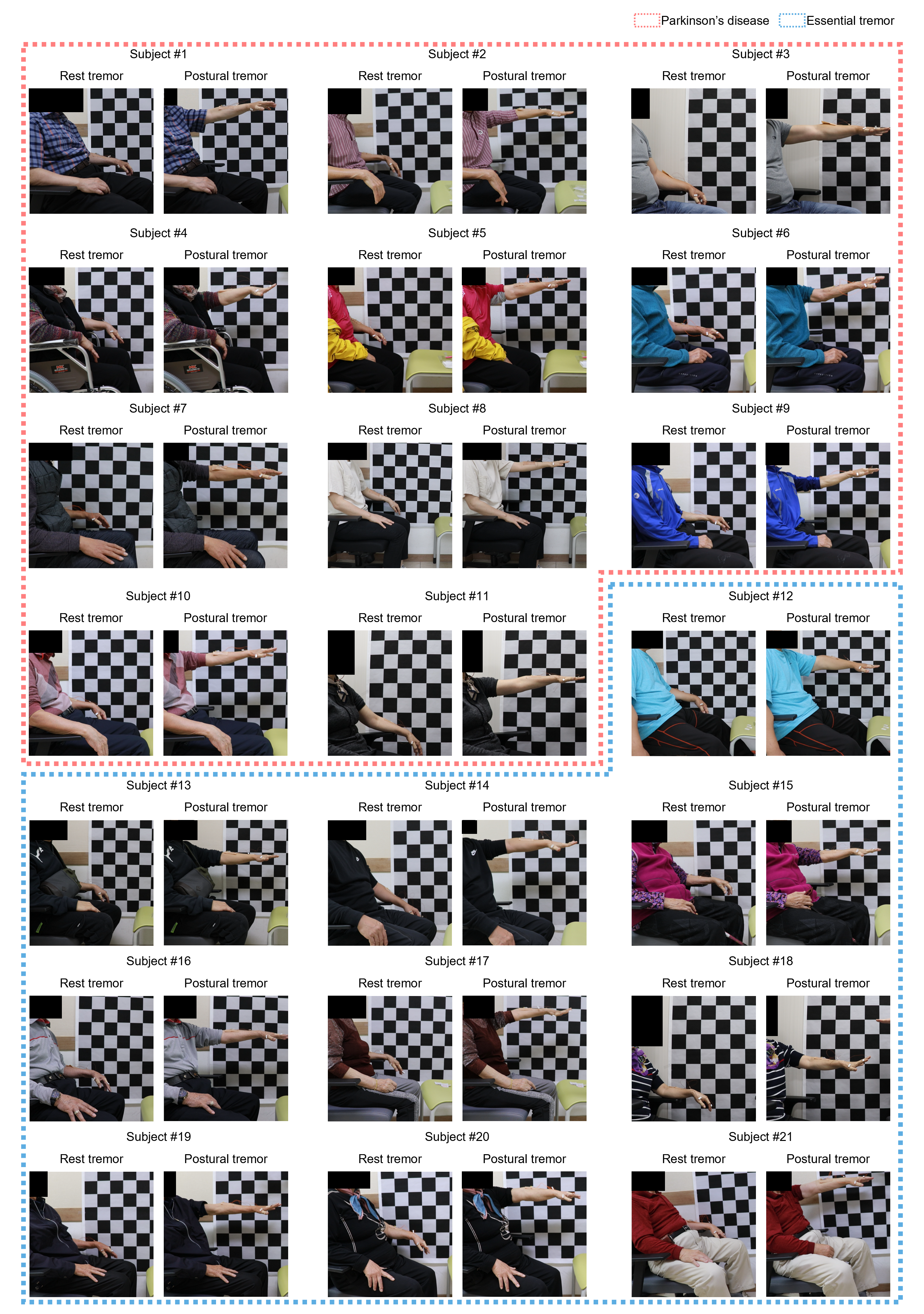


**Figure S18.** Photos of patients with Parkinson’s disease and Essential tremor in a clinical trial.


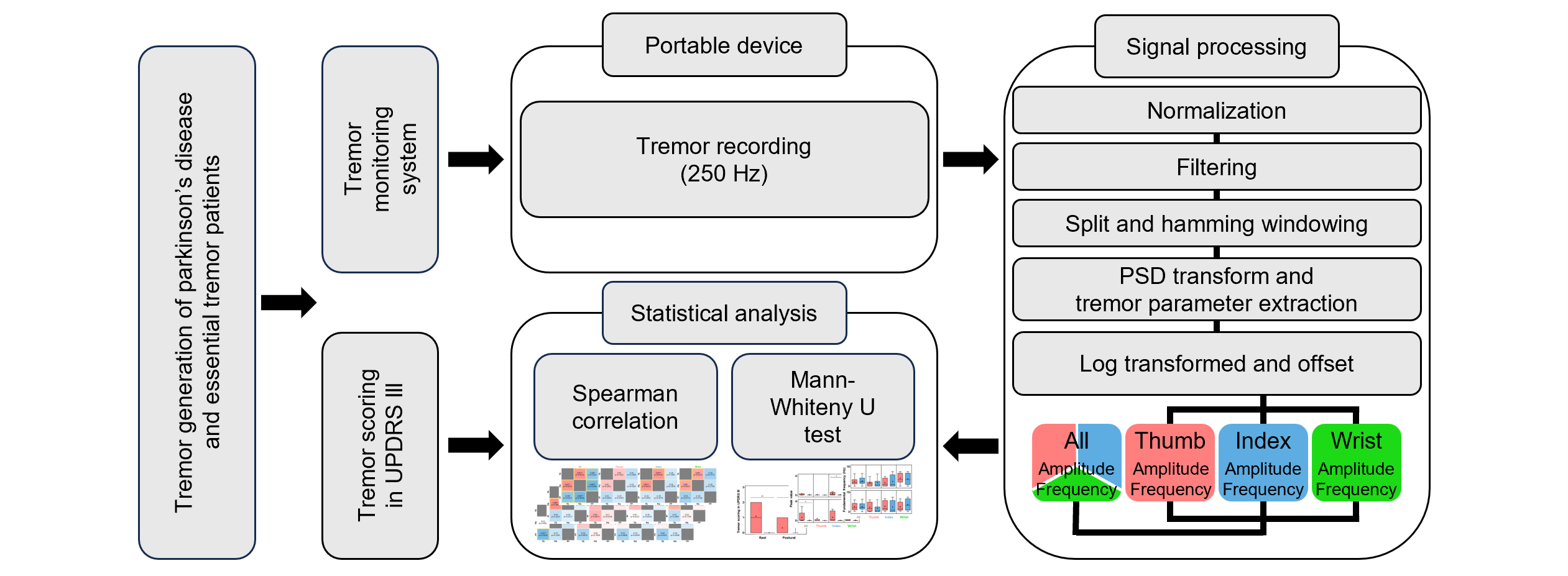


**Figure S19.** Schematic of tremor data analysis.

**Table S6.** Specification of patients (Parkinson’s disease and essential tremor)

Subject with Parkinson’s disease

| Group | Participants | Sex | Age | Diagnosis | H & Y stage | Symptom duration | Tremor laterality site | Medication status | Medications |
| --- | --- | --- | --- | --- | --- | --- | --- | --- | --- |
| PD | Subject#1 | M | 68 | Mixed-type PD | 2.5 | 10 years | Rt | ON | LD 100mg, PPX 1.125mg,  Procyclidine 2.5mg, RAS 1mg |
| PD | Subject#2 | F | 67 | TrD-PD | 2.5 | 8 years | Lt | ON | LD 550mg, PPX 0.125mg, TPM 25mg, baclofen 20mg, benztropine 1mg, tizanidine 3mg |
| PD | Subject#3 | M | 47 | TrD-PD | 1.5 | 2 years | Lt | ON | LD 150mg, PPX 1.125mg, RAS 1mg |
| PD | Subject#4 | F | 73 | TrD-PD | 2.5 | 6 years | Lt | ON | LD 500mg, PPX 0.25mg |
| PD | Subject#5 | F | 78 | TrD-PD | 1.5 | 3 years | Rt | ON | LD 350mg, PPX 0.125mg |
| PD | Subject#6 | M | 90 | TrD-PD | 2.5 | 0.5 years | Lt | ON | LD 450mg |
| PD | Subject#7 | M | 58 | TrD-PD with ET* | 2 | 4 years | Rt | ON | LD 525mg, PPX 1.125mg, procyclidine 10mg, BB 60mg, baclofen 30mg, |
| PD | Subject#8 | F | 47 | TrD-PD with ET* | 2 | 3 years | Rt | ON | PPX 0.75mg, RAS 1mg,  Trihexyphenidyl 1mg, BB 20mg |
| PD | Subject#9 | M | 69 | TrD-PD with ET | 1 | 5 years | Rt | ON | LD 500mg, BB 20mg, |
| PD | Subject#10 | M | 77 | TrD-PD with ET | 1.5 | 5 years | Rt | ON | LD 600mg, primidone 62.5mg, |
| PD | Subject#11 | F | 76 | TrD-PD with ET | 1 | 5 years | Rt | ON | LD 300mg, PPX 1mg, procyclidine 2.5mg, BB 20mg |

Abbreviation; LD, levodopa; PPX, pramipexole; BB, propranolol; RAS, rasagiline; TPM, Topamax; PD, Parkinson’s disease; ET, Essential tremor; TrD-PD, Tremor-dominant PD; H&Y, Hoehn & Yahr.

* Coexisting essential tremor was well controlled with medication; thus, only the resting tremor was observed at assessment.

Subject with Essential tremor

| Group | Participants | Sex | Age | Diagnosis | Treatment duration | Tremor laterality site | Medication status | Medications |
| --- | --- | --- | --- | --- | --- | --- | --- | --- |
| ET | Subject#12 | M | 45 | ET | 3.5 years | Lt | ON | BB 80mg, TPM 50mg, primidone 125mg, zonisamide 100mg, clonazepam 0.5mg |
| ET | Subject#13 | F | 41 | ET | 1.5 years | Rt | ON | BB 20mg, |
| ET | Subject#14 | M | 40 | ET | 3 years | Lt | ON | BB 20mg |
| ET | Subject#15 | F | 90 | ET | 3 years | Lt | OFF | No medication |
| ET | Subject#16 | M | 83 | ET | 3 years | Rt | ON | BB 20mg |
| ET | Subject#17 | F | 76 | ET | 3 years | Rt | ON | BB 80mg, alprazolam 0.25mg |
| ET | Subject#18 | F | 64 | ET | 3 years | Lt | ON | BB 80mg, TPM 25mg |
| ET | Subject#19 | M | 84 | ET | 0 year | Lt | OFF | No medication |
| ET | Subject#20 | F | 71 | ET | 0.5 year | Lt | ON | BB 20mg |
| ET | Subject#21 | M | 82 | ET | 0 year | Lt | OFF | No medication |

**Table S7.** Data distribution analysis between disease types and tremor types (Mann–Whitney U test, Significance level *p* < 0.01, *p* < 0.05)

| Indicator | Location | PD (rest) vs Baseline state | | | |
| --- | --- | --- | --- | --- | --- |
|  |  | *p*-value | U | HL estimate (95% CI) | N (PD/ET) |
| Tremor scoring in UPDRS III | - | 0.0037 | 15 | 1.0000  (0 to 2.0000) | 11/10 |
| Peak Amplitude | All | 0.0295 | 24 | 0.0636  (0.0159 to 1.5556) | 11/10 |
|  | Thumb | 0.0357 | 25 | 0.0070  (0.0002 to 0.2237) | 11/10 |
|  | Index | 0.0465 | 23 | 0.1280  (0.0003 to 2.0139) | 11/9 |
|  | Wrist | 0.0197 | 22 | 0.0744  (0.0029 to 0.8835) | 11/10 |
| Fundamental Frequency | All | 0.0513 | 26.5 | -2.6111  (-4.0303 to -0.1111) | 11/10 |
|  | Thumb | 0.0513 | 26.5 | -2.1667  (-5.0909 to -0.0909) | 11/10 |
|  | Index | 0.2014 | 31.5 | -3.0000  (-5.0000 to 1.0000) | 11/9 |
|  | Wrist | 0.2512 | 37.5 | -2.4167  (-5.2917 to 0.6000) | 11/10 |

| Indicator | Location | PD (postural) vs ET (postural) | | | |
| --- | --- | --- | --- | --- | --- |
|  |  | *p*-value | U | HL estimate (95% CI) | N (PD/ET) |
| Tremor scoring in UPDRS III | - | 0.0610 | 27.5 | -0.5000  (-1.0000 to 0) | 11/10 |
| Peak Amplitude | All | 0.0720 | 29 | 0.0354  (-0 to 0.2620) | 11/10 |
|  | Thumb | 0.1519 | 30 | 0.0017  (-0.0003 to 0.0121) | 11/9 |
|  | Index | 0.0357 | 25 | 0.0918  (0.0025 to 0.5282) | 11/10 |
|  | Wrist | 0.9177 | 53 | 0.0017  (-0.0198 to 0.0226) | 11/10 |
| Fundamental Frequency | All | 0.8094 | 51 | -0.2444  (-2.3333 to 1.3333) | 11/10 |
|  | Thumb | 1 | 48.5 | 0  (-3.0000 to 2.0000) | 11/9 |
|  | Index | 0.9725 | 54 | 0  (-4.0000 to 1.2000) | 11/10 |
|  | Wrist | 0.9177 | 52.5 | 0  (-4.0000 to 3.0000) | 11/10 |

| Indicator | Location | PD (rest) vs ET (postural) | | | |
| --- | --- | --- | --- | --- | --- |
|  |  | *p*-value | U | HL estimate (95% CI) | N (PD/ET) |
| Tremor scoring in UPDRS III | - | 0.5116 | 45 | 0  (-0.5000 to 1.0000) | 11/10 |
| Peak Amplitude | All | 0.0242 | 23 | 0.0674  (0.0241 to 1.5594) | 11/10 |
|  | Thumb | 0.0465 | 23 | 0.0090  (0.0001 to 0.2236) | 11/9 |
|  | Index | 0.0845 | 30 | 0.1255  (-0.0005 to 1.8800) | 11/10 |
|  | Wrist | 0.0513 | 27 | 0.0795  (0.0018 to 0.9088) | 11/10 |
| Fundamental Frequency | All | 0.9177 | 52.5 | -0.0540  (-2.0389 to 1.9610) | 11/10 |
|  | Thumb | 0.4561 | 38.5 | 0.3333  (-1.5000 to 3.0000) | 11/9 |
|  | Index | 0.5116 | 45 | 1.0000  (-2.1250 to 2.9375) | 11/10 |
|  | Wrist | 0.4262 | 42.5 | -1  (-6.0000 to 1.2500) | 11/10 |

Abbreviation; HL, Hodges–Lehmann; CI, confidence intervals.

* The 95% confidence intervals for Hodges–Lehmann estimates were calculated using percentile bootstrap resampling (10,000 iterations).

**Table S8.** Data correlation analysis Parkinson’s disease according to tremor type and location (Spearman correlation, Significance level *p* < 0.01, *p* < 0.05)

| Indicator | Location | Rest tremor | | | Postural tremor | | |
| --- | --- | --- | --- | --- | --- | --- | --- |
|  |  | *p*-value | Rho (95% CI) | N | P-value | Rho (95% CI) | N |
| Tremor scoring in UPDRS III vs  Peak Amplitude | All | 0.0016 | 0.8283  (0.4540 to 0.9541) | 11 | 0.9504 | 0.0213  (-0.5861 to 0.6133) | 11 |
|  | Thumb | 0.1621 | 0.4527  (-0.2021 to 0.8278) | 11 | 0.1909 | 0.4264  (-0.2331 to 0.8172) | 11 |
|  | Index | 0.0031 | 0.7994  (0.3834 to 0.9458) | 11 | 0.7551 | 0.1066  (-0.5270 to 0.6640) | 11 |
|  | Wrist | 0.0203 | 0.6838  (0.1423 to 0.9103) | 11 | 0.7551 | 0.1066  (-0.5270 to 0.6640) | 11 |
| Tremor scoring in UPDRS III vs  Fundamental Frequency | All | 0.0203 | -0.6838  (-0.9103 to -0.1423) | 11 | 0.0018 | -0.8246  (-0.9530 to -0.4447) | 11 |
|  | Thumb | 0.206 | -0.4136  (-0.8120 to 0.2478) | 11 | 0.0878 | -0.5380  (-0.8602 to 0.0914) | 11 |
|  | Index | 0.3907 | -0.2878  (-0.7570 to 0.3772) | 11 | 0.0808 | -0.5482  (-0.8640 to 0.0770) | 11 |
|  | Wrist | 0.0733 | -0.5599  (-0.8682 to 0.0602) | 11 | 0.0505 | -0.6011  (-0.8827 to -0.0019) | 11 |
| Peak Amplitude vs Fundamental Frequency | All | 0.0006 | -0.8636  (-0.9640 to -0.5472) | 11 | 0.3165 | -0.3333  (-0.7777 to 0.3332) | 11 |
|  | Thumb | 0.4563 | -0.2511  (-0.7396 to 0.4106) | 11 | 0.7906 | 0.0908  (-0.5384 to 0.6550) | 11 |
|  | Index | 0.3678 | -0.3014  (-0.7633 to 0.3644) | 11 | 0.4626 | -0.2478  (-0.7380 to 0.4136) | 11 |
|  | Wrist | 0.2769 | -0.3599  (-0.7894 to 0.3061) | 11 | 0.4468 | -0.2563  (-0.7421 to 0.4060) | 11 |

Abbreviation; CI, confidence intervals.

* The 95% confidence intervals for Spearman’s rank correlation coefficient were calculated using Fisher’s z transformation.

**Table S9.** Data correlation analysis Essential tremor according to tremor type and location (Spearman correlation, Significance level *p* < 0.01, *p* < 0.05)

| Indicator | Location | | Rest tremor | | | | Postural tremor | | | |
| --- | --- | --- | --- | --- | --- | --- | --- | --- | --- | --- |
|  |  | | *p*-value | Rho (95% CI) | N | | P-value | Rho (95% CI) | N |  |
| Tremor scoring in UPDRS III vs  Peak Amplitude | | All | | - | - | - | | 0.0007 | 0.8842  (0.5743 to 0.9724) | 10 |
|  |  | Thumb | | - | - | - | | 0.1627 | 0.5080  (-0.1788 to 0.8619) | 10 |
|  |  | Index | | - | - | - | | 0.0908 | 0.5621  (-0.1628 to 0.8929) | 9 |
|  |  | Wrist | | - | - | - | | 0.0414 | 0.6512  (0.0366 to 0.9084) | 10 |
| Tremor scoring in UPDRS III vs  Fundamental Frequency | | All | | - | - | - | | 0.3057 | -0.3608  (-0.8071 to 0.3479) | 10 |
|  |  | Thumb | | - | - | - | | 0.0950 | 0.5892  (-0.0643 to 0.8890) | 10 |
|  |  | Index | | - | - | - | | 0.9279 | 0.0330  (-0.6453 to 0.6822) | 9 |
|  |  | Wrist | | - | - | - | | 0.2877 | -0.3735  (-0.8121 to 0.3349) | 10 |
| Peak Amplitude vs Fundamental Frequency | | All | | - | - | - | | 0.5282 | -0.2270  (-0.7495 to 0.4698) | 10 |
|  |  | Thumb | | - | - | - | | 0.6044 | 0.2008  (-0.4909 to 0.7372) | 10 |
|  |  | Index | | - | - | - | | 0.7480 | -0.1168  (-0.7247 to 0.5934) | 9 |
|  |  | Wrist | | - | - | - | | 0.6983 | 0.1407  (-0.5365 to 0.7076) | 10 |

Abbreviation; CI, confidence intervals.

* The 95% confidence intervals for Spearman’s rank correlation coefficient were calculated using Fisher’s z transformation.

**Table S10.** Data correlation analysis of Parkinson’s disease according to tremor type and location after multiple-comparison correction (Spearman correlation, Multiple-comparison correction: Benjamini-Hochberg method, Significance level *p* < 0.01, *p* < 0.05)

| Indicator | Location | Rest tremor | | | Postural tremor | | |
| --- | --- | --- | --- | --- | --- | --- | --- |
|  |  | *p*-value | Rho (95% CI) | N | P-value | Rho (95% CI) | N |
| Tremor scoring in UPDRS III vs  Peak Amplitude | All | 0.0096 | 0.8283  (0.4540 to 0.9541) | 11 | 0.9504 | 0.0213  (-0.5861 to 0.6133) | 11 |
|  | Thumb | 0.2279 | 0.4527  (-0.2021 to 0.8278) | 11 | 0.4582 | 0.4264  (-0.2331 to 0.8172) | 11 |
|  | Index | 0.0124 | 0.7994  (0.3834 to 0.9458) | 11 | 0.8625 | 0.1066  (-0.5270 to 0.6640) | 11 |
|  | Wrist | 0.0487 | 0.6838  (0.1423 to 0.9103) | 11 | 0.8625 | 0.1066  (-0.5270 to 0.6640) | 11 |
| Tremor scoring in UPDRS III vs  Fundamental Frequency | All | 0.0487 | -0.6838  (-0.9103 to -0.1423) | 11 | 0.0216 | -0.8246  (-0.9530 to -0.4447) | 11 |
|  | Thumb | 0.309 | -0.4136  (-0.8120 to 0.2478) | 11 | 0.2634 | -0.5380  (-0.8602 to 0.0914) | 11 |
|  | Index | 0.4262 | -0.2878  (-0.7570 to 0.3772) | 11 | 0.2634 | -0.5482  (-0.8640 to 0.0770) | 11 |
|  | Wrist | 0.1466 | -0.5599  (-0.8682 to 0.0602) | 11 | 0.2634 | -0.6011  (-0.8827 to -0.0019) | 11 |
| Peak Amplitude vs Fundamental Frequency | All | 0.0072 | -0.8636  (-0.9640 to -0.5472) | 11 | 0.6330 | -0.3333  (-0.7777 to 0.3332) | 11 |
|  | Thumb | 0.4563 | -0.2511  (-0.7396 to 0.4106) | 11 | 0.8625 | 0.0908  (-0.5384 to 0.6550) | 11 |
|  | Index | 0.4262 | -0.3014  (-0.7633 to 0.3644) | 11 | 0.6939 | -0.2478  (-0.7380 to 0.4136) | 11 |
|  | Wrist | 0.3692 | -0.3599  (-0.7894 to 0.3061) | 11 | 0.6939 | -0.2563  (-0.7421 to 0.4060) | 11 |

Abbreviation; CI, confidence intervals.

* The 95% confidence intervals for Spearman’s rank correlation coefficient were calculated using Fisher’s z transformation.

**Table S11.** Data correlation analysis of Essential tremor according to tremor type and location after multiple-comparison correction (Spearman correlation, Multiple-comparison correction: Benjamini-Hochberg method, Significance level *p* < 0.01, *p* < 0.05)

| Indicator | Location | | Rest tremor | | | | Postural tremor | | | |
| --- | --- | --- | --- | --- | --- | --- | --- | --- | --- | --- |
|  |  | | *p*-value | Rho (95% CI) | N | | P-value | Rho (95% CI) | N |  |
| Tremor scoring in UPDRS III vs  Peak Amplitude | | All | | - | - | - | | 0.0084 | 0.8842  (0.5743 to 0.9724) | 10 |
|  |  | Thumb | | - | - | - | | 0.3905 | 0.5080  (-0.1788 to 0.8619) | 10 |
|  |  | Index | | - | - | - | | 0.2850 | 0.5621  (-0.1628 to 0.8929) | 9 |
|  |  | Wrist | | - | - | - | | 0.2484 | 0.6512  (0.0366 to 0.9084) | 10 |
| Tremor scoring in UPDRS III vs  Fundamental Frequency | | All | | - | - | - | | 0.5241 | -0.3608  (-0.8071 to 0.3479) | 10 |
|  |  | Thumb | | - | - | - | | 0.2850 | 0.5892  (-0.0643 to 0.8890) | 10 |
|  |  | Index | | - | - | - | | 0.9279 | 0.0330  (-0.6453 to 0.6822) | 9 |
|  |  | Wrist | | - | - | - | | 0.5241 | -0.3735  (-0.8121 to 0.3349) | 10 |
| Peak Amplitude vs Fundamental Frequency | | All | | - | - | - | | 0.7923 | -0.2270  (-0.7495 to 0.4698) | 10 |
|  |  | Thumb | | - | - | - | | 0.8059 | 0.2008  (-0.4909 to 0.7372) | 10 |
|  |  | Index | | - | - | - | | 0.8160 | -0.1168  (-0.7247 to 0.5934) | 9 |
|  |  | Wrist | | - | - | - | | 0.8160 | 0.1407  (-0.5365 to 0.7076) | 10 |

Abbreviation; CI, confidence intervals.

* The 95% confidence intervals for Spearman’s rank correlation coefficient were calculated using Fisher’s z transformation.


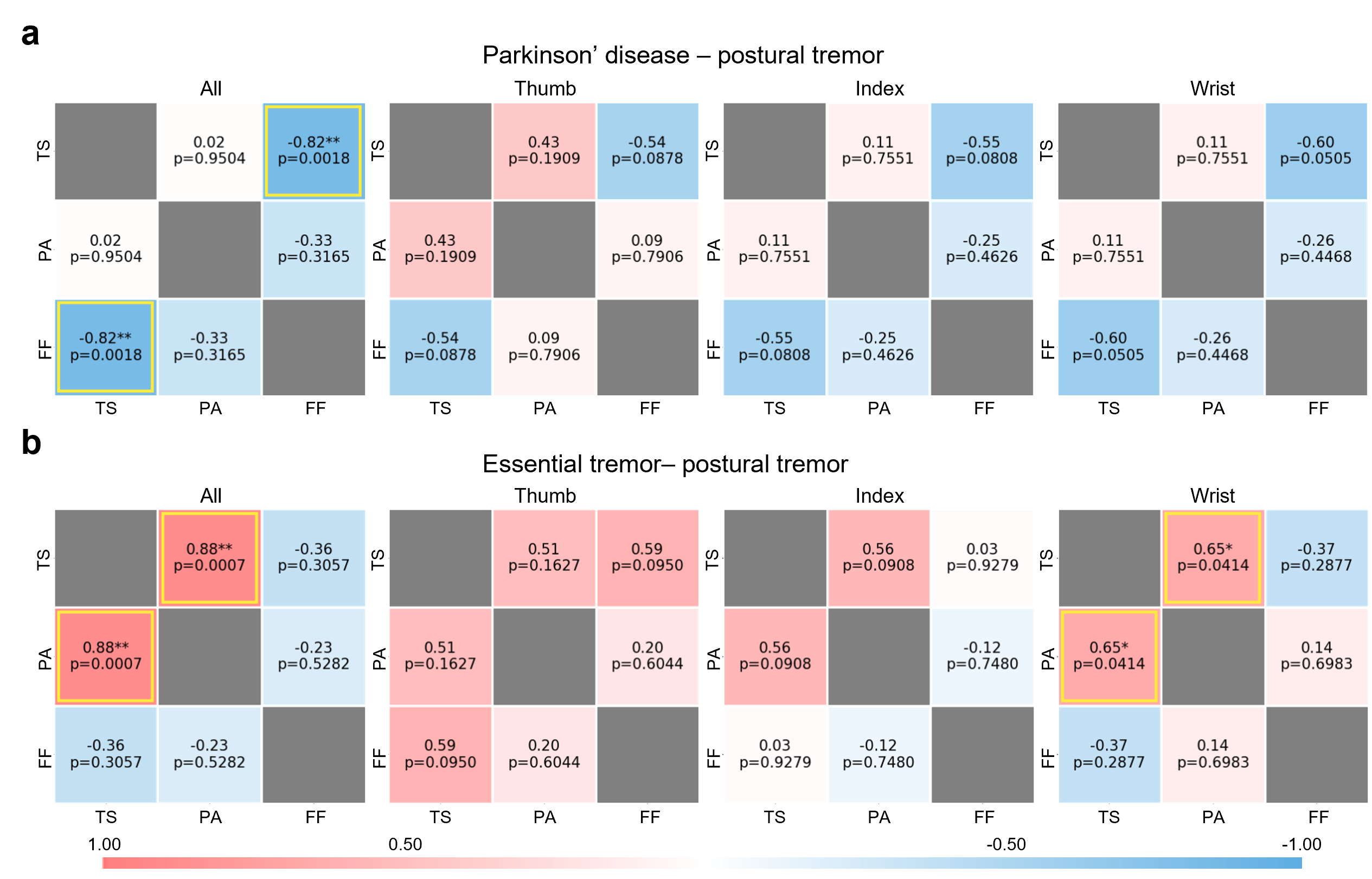


**Figure S20.** Spearman correlation heatmap between tremor score in UPDRS III (UPDRS

), Peak Amplitude (PA), Fundamental Frequency (FF) across all, thumb, index, wrist under postural tremor in (a) Parkinson’s disease patients and (b) Essential tremor patients. (** is *p* < 0.01, * is *p* < 0.05)

**References**

1. Y. Du, Y. Liu, W. Lu, X. Zhang, A. Wang, J. Kong, “Nacre-Inspired MXene Nanocomposite-based Strain Sensor with Ultrahigh Sensitivity in a Small Strain Range for Parkinson's Disease Diagnosis,” *ACS Applied Materials & Interfaces* 15, no.43 (2023): 50413–50426.

https://doi.org/10.1021/acsami.3c13815

2. X. Wang, W. Fu, G. Gao, et al., “Self-cross-linked arrays enabled flexible mechanical sensors for monitoring the body tremor,” *npj Flexible Electronics* 4, no.1 (2020): 8.

https://doi.org/10.1038/s41528-020-0071-3

3. D. Kang, P. V. Pikhitsa, Y. W. Choi, et al., “Ultrasensitive mechanical crack-based sensor inspired by the spider sensory system,” *Nature* 516, no.7530 (2014): 222–226.

https://doi.org/10.1038/nature14002

4. T. Lee, Y. W. Choi, G. Lee, S. M. Kim, D. Kang, M. Choi, “Crack-based strain sensor with diverse metal films by inserting an inter-layer,” *RSC Advances* 7, no.55 (2017): 34810–34815.

https://doi.org/10.1039/c7ra05837c

5. C. Zhang, J. Sun, Y. Lu, J. Liu, “Nanocrack-based strain sensors,” *Journal of Materials Chemistry C* 9, no.3 (2021): 754–772.

https://doi.org/10.1039/d0tc04346j

6. W. Wang, Y. Liu, M. Ding, et al., “From network to channel: Crack-based strain sensors with high sensitivity, stretchability, and linearity via strain engineering,” *Nano Energy* 116, (2023): 108832.

https://doi.org/10.1016/j.nanoen.2023.108832

7. S. Soltanian, R. Rahmanian, B. Gholamkhass, N. M. Kiasari, F. Ko, P. Servati, “Highly Stretchable, Sparse, Metallized Nanofiber Webs as Thin, Transferrable Transparent Conductors,” *Advanced Energy Materials* 3, no.10 (2013): 1332–1337.

https://doi.org/10.1002/aenm.201300193

8. Y. Bai, Y. Zhou, X. Wu, et al., “Flexible Strain Sensors with Ultra-High Sensitivity and Wide Range Enabled by Crack-Modulated Electrical Pathways,” *Nano-Micro Letters* 17, no.1 (2025): 64.

https://doi.org/10.1007/s40820-024-01571-6

9. Y. Wang, S. Lee, T. Yokota, et al., “A durable nanomesh on-skin strain gauge for natural skin motion monitoring with minimum mechanical constraints,” *Science Advances* 6, no.33 (2020): eabb7043.

https://doi.org/10.1126/sciadv.abb7043

10. X. Xie, G. Liu, H. Li, W. Yuan, S. Guo, “A conformable, durable, adhesive welded fiber mate for on-skin strain sensing,” *Chemical Engineering Journal* 457, (2023): 141233.

https://doi.org/10.1016/j.cej.2022.141233

11. G. Liu, Y. Zhang, B. Li, W. Yuan, C. Meng, S. Guo, “A highly sensitive stretchable constraint fiber mat sensor with tailored near-zero Poisson's ratio for very early stage of battery over charge/ overdischarge monitoring,” *Chemical Engineering Journal* 490, (2024): 151612.

https://doi.org/10.1016/j.cej.2024.151612

12. N. Z. Al-Hazeem, “Effect of the Distance between the Needle Tip and the Collector on Nanofibers Morphology,” *Nanomedicine & Nanotechnology Open Access* 5, no.3 (2020): 1–5.

https://doi.org/10.23880/nnoa-16000195

13. K. Chen, S. P. Nikam, Z. K. Zander, et al., “Continuous Fabrication of Antimicrobial Nanofiber Mats Using Post-Electrospinning Functionalization for Roll-to-Roll Scale-Up,” *ACS Applied Polymer Materials* 2, no.2 (2020): 304–316.

https://doi.org/10.1021/acsapm.9b00798

14. M. Dasdemir, M. Topalbekiroglu, A. Demir, “Electrospinning of thermoplastic polyurethane microfibers and nanofibers from polymer solution and melt,” *Journal of Applied Polymer Science* 127, no.3 (2013): 1901–1908.

https://doi.org/10.1002/app.37503

15. D. Wang, X. Li, H. Tian, et al., “Flexible strain sensor based on embedded three-dimensional annular cracks with high mechanical robustness and high sensitivity,” *Applied Materials Today* 25, (2021): 101247.

https://doi.org/10.1016/j.apmt.2021.101247

16. Y. Zhao, M. Ren, Y. Shang, et al., “Ultra-sensitive and durable strain sensor with sandwich structure and excellent anti-interference ability for wearable electronic skins,” *Composites Science and Technology* 200, (2020): 108448.

https://doi.org/10.1016/j.compscitech.2020.108448

17. S. Kim, J. Kang, I. Lee, et al., “An intrinsically stretchable multi-biochemical sensor for sweat analysis using photo-patternable ecoflex,” *npj Flexible Electronics* 7, no.1 (2023): 33.

https://doi.org/10.1038/s41528-023-00268-x

18. M. Fortunato, I. Bellagamba, A. Tamburrano, M. S. Sarto, “Flexible Ecoflex**^®^**

/Graphene Nanoplatelet Foams for Highly Sensitive Low-Pressure Sensors,” *Sensors* 20, no.16 (2020): 4406.

https://doi.org/10.3390/s20164406
